# Distinct epigenetic signatures between adult-onset and late-onset depression

**DOI:** 10.1038/s41598-021-81758-8

**Published:** 2021-01-27

**Authors:** Hirotaka Yamagata, Hiroyuki Ogihara, Koji Matsuo, Shusaku Uchida, Ayumi Kobayashi, Tomoe Seki, Masaaki Kobayashi, Kenichiro Harada, Chong Chen, Shigeo Miyata, Masato Fukuda, Masahiko Mikuni, Yoshihiko Hamamoto, Yoshifumi Watanabe, Shin Nakagawa

**Affiliations:** 1 Division of Neuropsychiatry, Department of Neuroscience, Yamaguchi University Graduate School of Medicine, 1-1-1 Minami-kogushi, Ube, Yamaguchi 755-8505 Japan; 2 Core Research for Evolutional Science and Technology (CREST), Japan Science and Technology Agency (JST), 4-1-8 Honcho, Kawaguchi, Saitama 332-0012 Japan; 3 Division of Electrical, Electronic and Information Engineering, Graduate School of Sciences and Technology for Innovation, Yamaguchi University, 2-16-1 Tokiwadai, Ube, Yamaguchi 755-8611 Japan; 4 Department of Genetic and Behavioral Neuroscience, Gunma University Graduate School of Medicine, 3-39-22 Showa-machi, Maebashi, Gunma 371-8511 Japan; 5 Department of Psychiatry and Neuroscience, Gunma University Graduate School of Medicine, 3-39-22 Showa-machi, Maebashi, Gunma 371-8511 Japan; 6 Hakodate Watanabe Hospital, 1-31-1 Yunokawacho, Hakodate, Hokkaido 042-8678 Japan; 7 Present Address: Department of Psychiatry, Faculty of Medicine, Saitama Medical University, 38 Morohongo, Moroyama, Iruma, Saitama 350-0495 Japan; 8 Present Address: SK Project, Medical Innovation Center, Kyoto University Graduate School of Medicine, 53 Shogoin-Kawahara-cho, Sakyo-ku, Kyoto, 606-8507 Japan; 9 Present Address: Department of Psychiatry, Hokkaido University, Graduate School of Medicine, Kita 15, Nishi 7, Kita-ku, Sapporo, 060-8638 Japan; 10 Present Address: Southern TOHOKU Research Institute for Neuroscience, Southern TOHOKU General Hospital, 7-115 Yatsuyamada, Koriyama, Fukushima 963-8052 Japan

**Keywords:** Diagnostic markers, Depression, DNA methylation

## Abstract

The heterogeneity of major depressive disorder (MDD) is attributed to the fact that diagnostic criteria (e.g., DSM-5) are only based on clinical symptoms. The discovery of blood biomarkers has the potential to change the diagnosis of MDD. The purpose of this study was to identify blood biomarkers of DNA methylation by strategically subtyping patients with MDD by onset age. We analyzed genome-wide DNA methylation of patients with adult-onset depression (AOD; age ≥ 50 years, age at depression onset < 50 years; N = 10) and late-onset depression (LOD; age ≥ 50 years, age at depression onset ≥ 50 years; N = 25) in comparison to that of 30 healthy subjects. The methylation profile of the AOD group was not only different from that of the LOD group but also more homogenous. Six identified methylation CpG sites were validated by pyrosequencing and amplicon bisulfite sequencing as potential markers for AOD in a second set of independent patients with AOD and healthy control subjects (N = 11). The combination of three specific methylation markers achieved the highest accuracy (sensitivity, 64%; specificity, 91%; accuracy, 77%). Taken together, our findings suggest that DNA methylation markers are more suitable for AOD than for LOD patients.

## Introduction

Depressive disorders are among the most common psychiatric diseases. However, it is often difficult to discriminate major depressive disorder (MDD) from other depressive disorders based on operational diagnostic criteria (e.g., Diagnostic And Statistical Manual of Mental Disorders, Fifth Edition [DSM-5]) because these criteria mainly rely on patients’ symptoms and do not include laboratory findings. Moreover, MDD is probably a heterogeneous entity because the pathophysiology of depression includes multiple factors such as genetics and environment^1^. The onset age is a key factor to heterogeneity in various diseases, for example, diabetes mellitus^2^ and asthma^3^. Systematic reviews reported that patients with late-onset depression (LOD) have a lower frequency of family history^4^, decrease in cognitive executive function^5^, and more frequent and pronounced white matter hyperintensities in magnetic resonance imaging^6^ compared to patients with early-onset depression, suggesting different pathophysiologies. Recently, we discovered a validated gene expression marker for MDD in patients aged 50 years or older with confirmed MDD diagnosis, without a manic episode, and onset before the age of 50 years^7^. Other reports also suggest heterogeneity of MDD because leukocytic gene-expression profiles differ between adult-onset depression (AOD) and LOD^7,8^.

DNA methylation is one of the most common epigenetic modifications, controlling gene expression^9,10^. DNA methylation is known to control the resilience to early life stress and depression-like behavior in mice^11^. In humans, several reports show changed DNA methylation in the peripheral blood of patients with depression^12,13^. As an indicative result, we reported alterations in mRNAs of DNA methyltransferases^14^. However, few studies have focused on stratifying MDD by DNA methylation.

We postulate that epigenetic factors have a more significant influence on AOD than on LOD. The aim of this study was the analysis of the differences in methylation profiles of patients with AOD and LOD. We also identified DNA methylation markers from probes of patients with AOD.

## Results

The demographic and clinical data of Group 1a (discovery samples) are shown in Table 1a. Participants with AOD were significantly younger than those in the healthy control (HC) group. The mean percentage of neutrophils was higher in the LOD than the HC group.

**Table 1:** Participants’ demographics and characteristics in Group 1a and 1b (discovery and training samples, respectively). Characteristics of the participants for the genome-wide methylation array (**a**) and for the validation using pyrosequencing and amplicon bisulfite sequencing (**b**). Statistical analyses performed using the EZR software (version 1.37) (http://www.jichi.ac.jp/saitama-sct/SaitamaHP.files/statmedEN.html). Data are shown as the mean ± standard deviation. *p < 0.05 compared to HC. ^#^p < 0.05 compared to AOD.

	HC	AOD	LOD
**(a)**			
No. of subjects	30	10	25
Sex (Male/Female)	13/17	5/5	9/16
Age (years)	63.3 ± 8.0	56.0 ± 6.8*	61.8 ± 7.5
Age at onset (years)	–	35.7 ± 8.9	57.8 ± 6.7^#^
Total duration of all MDD episodes (months)	–	7.0 ± 6.4	13.2 ± 16.4
Number of episodes	–	3.0 ± 1.7	2.1 ± 2.1
Number of WBCs	4928 ± 1430	4757 ± 1495	5109 ± 1773
Neutrophils (%)	58.1 ± 8.7	58.4 ± 9.1	63.9 ± 6.6*
Lymphocytes (%)	33.2 ± 8.1	33.1 ± 7.5	28.3 ± 6.4
Eosinophils (%)	3.0 ± 1.7	3.0 ± 2.1	2.4 ± 2.0
Basophils (%)	0.45 ± 0.25	0.51 ± 0.29	0.55 ± 0.53
Monocytes (%)	5.2 ± 1.3	5.0 ± 1.5	4.8 ± 1.0
SIGH-D	0.7 ± 0.9	21.7 ± 2.2*	22.4 ± 4.3*
Equivalent dose of imipramine (mg)	–	221 ± 131	183 ± 147
**(b)**			
No. of subjects	10	10	10
Sex (Male/Female)	5/5	5/5	5/5
Age (years)	59.7 ± 6.2	56.0 ± 6.8	59.2 ± 4.6
Age at onset (years)	–	35.7 ± 8.9	55.5 ± 4.5^#^
Total duration of all MDD episodes (months)	–	7.0 ± 6.4	21.8 ± 22.5
Number of episodes	–	3.0 ± 1.7	2.3 ± 2.5
Number of WBCs	5171 ± 1180	4757 ± 1495	5333 ± 1667
Neutrophils (%)	59.9 ± 9.0	58.4 ± 9.1	60.5 ± 6.3
Lymphocytes (%)	32.3 ± 8.8	33.1 ± 7.5	32.3 ± 5.7
Eosinophils (%)	2.4 ± 1.7	3.0 ± 2.1	1.9 ± 2.0
Basophils (%)	0.48 ± 0.28	0.51 ± 0.29	0.42 ± 0.30
Monocytes (%)	4.9 ± 1.5	5.0 ± 1.5	4.9 ± 1.1
SIGH-D	0.7 ± 0.7	21.7 ± 2.2*	23.0 ± 4.7*
Equivalent dose of imipramine (mg)	–	221 ± 131	218 ± 198

AOD, adult-onset depression; HC, healthy controls; LOD, late-onset depression; MDD, major depressive disorder; SIGH-D, Structured Interview Guide of the Hamilton Depression Rating Scale score; WBCs, white blood cells.

We identified specific methylation sites associated with AOD and LOD compared to HC from 411,383 probes using the Fisher ratio (F-ratio), which compares the variance between classes relative to the variance within classes. An increase in the F-ratio generally suggests that a feature distinguishes better between compared classes. The comparison between the top-ranked probes of the AOD and LOD groups and the HC group are shown in Table 2 and Supplementary Table S1. The F-ratios of the top-ranked probes distinguishing the AOD from the HC group were higher than those discriminating between the LOD and HC groups. Similarly, the F-ratios of the top-ranked RefSeq genes differentiating the AOD from the HC group were also higher than those distinguishing the LOD from the HC group (Supplementary Table S2).

**Table 2:** The 20 top-ranked DNA methylation sites in the AOD versus HC group (**a**) and the LOD versus HC group (**b**).

ID	Fisher Ratio	HC average	AOD average	CHR	MAPINFO	UCSC_REFGENE_NAME	UCSC_REFGENE_GROUP	RELATION_TO_UCSC_CPG_ISLAND
**(a)**								
cg15794987	3.85	0.954	0.937	8	6,534,897			
cg07763047	3.71	0.046	0.025	5	160974837	GABRB2;GABRB2	5′UTR;5′UTR	Island
cg21347377	3.59	0.014	0.006	10	96,305,691	HELLS	1stExon	Island
cg17458347	3.53	0.099	0.083	1	42,928,946			Island
cg14567489	3.41	0.021	0.014	20	43,977,078	SDC4	TSS200	Island
cg02221310	3.33	0.013	0.004	13	41,363,578	SLC25A15;SLC25A15	5′UTR;1stExon	Island
cg16579770	3.29	0.934	0.917	16	72,058,938	DHODH	3′UTR	
cg01320648	3.22	0.548	0.517	4	123091375	KIAA1109	TSS1500	
cg05974695	3.19	0.026	0.017	11	130786536	SNX19	TSS200	Island
cg15525503	3.13	0.020	0.008	1	3,773,166	DFFB;KIAA0562	TSS1500;5′UTR	N_Shore
cg26655295	3.08	0.045	0.034	2	677,162	TMEM18	Body	Island
cg12867237	3.07	0.146	0.128	11	44,087,540	ACCS;ACCS	TSS200;TSS1500	Island
cg16234726	3.06	0.937	0.922	14	102099176			N_Shore
cg20452738	3.04	0.922	0.947	1	226828650	ITPKB	Body	N_Shore
cg23543123	2.99	0.023	0.013	4	48,485,569	SLC10A4;SLC10A4	5′UTR;1stExon	Island
cg22115465	2.91	0.983	0.979	7	2,170,343	MAD1L1;MAD1L1;MAD1L1	Body;Body;Body	
cg04147027	2.90	0.030	0.022	6	82,462,442	FAM46A	TSS200	Island
cg15971980	2.89	0.571	0.676	6	150254442			
cg24934561	2.89	0.064	0.055	1	3,663,902	KIAA0495	TSS200	Island
cg23531049	2.81	0.845	0.892	15	42,075,277	MAPKBP1;MAPKBP1	Body;Body	

ID	Fisher Ratio	HC average	LOD average	CHR	MAPINFO	UCSC_REFGENE_NAME	UCSC_REFGENE_GROUP	RELATION_TO_UCSC_CPG_ISLAND
**(b)**								
cg06557376	3.14	0.959	0.949	17	8,384,607	MYH10	Body	
cg23854456	2.87	0.936	0.920	19	39,806,075	LRFN1	TSS200	S_Shore
cg09597022	2.46	0.884	0.855	6	33,288,332	DAXX;DAXX;DAXX;DAXX	Body;Body;Body;Body	N_Shore
cg04521026	2.21	0.958	0.948	2	240067196	HDAC4	Body	
cg15390741	2.06	0.986	0.976	1	51,822,535	EPS15;EPS15	Body;Body	
cg06034437	2.03	0.521	0.474	3	10,183,196	VHL;VHL	TSS200;TSS200	N_Shore
cg25996553	1.99	0.889	0.869	8	13,133,084	DLC1	Body	N_Shore
cg18394552	1.92	0.648	0.577	5	159428643			
cg04262179	1.89	0.975	0.971	1	178482030	C1orf49;C1orf49;C1orf49;C1orf49	TSS200;TSS200;TSS200;TSS200	
cg03473408	1.88	0.769	0.696	6	30,588,689	MRPS18B	Body	S_Shelf
cg27318635	1.83	0.949	0.937	16	3,781,749	CREBBP;CREBBP	Body;Body	S_Shore
cg16011480	1.78	0.151	0.139	10	97,667,594	C10orf131	TSS200	Island
cg27088822	1.75	0.799	0.752	12	121731226	CAMKK2;CAMKK2;CAMKK2;CAMKK2;CAMKK2;CAMKK2;CAMKK2	5′UTR;5′UTR;5′UTR;5′UTR;5′UTR;5′UTR;5′UTR	N_Shelf
cg13081381	1.72	0.480	0.502	13	82,264,192			
cg01345727	1.71	0.759	0.695	13	52,734,018	NEK3;NEK3;NEK3;NEK3	TSS1500;TSS200;TSS200;TSS200	S_Shore
cg11855759	1.70	0.205	0.234	17	9,548,802	USP43	TSS200	Island
cg22389270	1.70	0.263	0.244	8	101162769	POLR2K	TSS200	
cg11558107	1.69	0.932	0.912	15	60,700,124			
cg20420249	1.66	0.808	0.758	1	172378938	DNM3;DNM3	3′UTR;3′UTR	
cg15587947	1.66	0.861	0.844	19	10,220,948	PPAN-P2RY11;P2RY11;PPAN	Body;TSS1500;Body	Island

AOD, adult-onset depression; HC, healthy controls; LOD, late-onset depression.

To validate the methylation levels of these probe sites determined by the genome-wide DNA methylation array, we analyzed some identified sites of Group 1b participants using pyrosequencing. The demographic and clinical data of Group 1b are shown in Table 1b. The sensitivity to detect methylation by pyrosequencing is about 5%^15^. Therefore, we chose the four probes cg15971980, cg07584066, cg20903900, and cg10294474 whose averaged methylation levels in the AOD group were significantly elevated by at least 5% compared to those in the HC group. The methylation levels of these sites determined by pyrosequencing significantly correlated with those determined by the methylation beads array. The correlation coefficients between the methylation levels of beads array and pyrosequencing, p-values, and scatter plots are shown in Supplementary Table S3a and Fig. S1, respectively. These results indicated that the findings of the methylation beads array were valid.

However, many of the target methylation sites with a high discrimination rate between AOD and HC presented only small differences of less than 5% in the methylation beads array analysis (Table 2). To validate small differences in methylation levels, we analyzed them additionally by amplicon bisulfite sequencing. We checked the six identified probes cg15794987, cg07763047, cg21347377, cg17458347, cg14567489, and cg16579770. The site cg15971980 was used as the positive control of the amplicon bisulfite sequencing procedure. The methylation levels in the amplicon bisulfite sequencing of the two sites cg07763047 and cg16579770 were significantly correlated with those in the methylation beads array (Supplementary Table S3b). Scatter plots demonstrate the relationships between clinical parameters and each methylation level (Supplementary Figs. S2-7). The methylation levels of the cg07763047 and cg10294474 sites correlated with the age at onset (Supplementary Table S4).

We focused on discriminating between the AOD and HC groups, while the methylation levels from Group 1b were used as the training sample. Each receiver operating characteristic (ROC) curve of the six cytosine methylation sites cg15971980, cg07584066, cg20903900, cg10294474, cg07763047, and cg16579770 in Group 1b was obtained by calculating their sensitivity and specificity. All values for cut-off point, sensitivity, specificity, and discrimination accuracy are displayed in Table 3. All six cytosine methylation sites demonstrated a high discrimination accuracy (≥ 70%).

**Table 3:** Sensitivity, specificity, and accuracy in the independent second set (Group 2) for discrimination between AOD and HC. The optimal cut-off points of Group 1b (training samples) were determined by maximizing the Youden index. Sensitivity, specificity, and discrimination accuracy for Group 2 (independent test samples) were calculated.

Probe ID	Cut-off points	Performance for training samples			Performance for test samples		
		Sensitivity	Specificity	Accuracy	Sensitivity	Specificity	Accuracy
cg07584066	0.308 >	0.70	0.70	0.70	0.82	0.73	0.77
cg07763047	0.018 >	1.00	0.40	0.70	0.91	0.46	0.68
cg10294474	0.789 <	1.00	0.60	0.80	0.55	0.36	0.46
cg15971980	0.691 <	0.90	0.80	0.85	0.36	0.46	0.41
cg16579770	0.889 >	0.80	0.70	0.75	0.73	0.64	0.68
cg20903900	0.828 >	1.00	0.50	0.75	0.55	0.73	0.64

AOD, adult-onset depression; HC, healthy controls.

Next, we employed a different, independent data set from Group 2 (test sample) to validate the above established cut-off points in discriminating between AOD and HC participants. The demographic and clinical data of Group 2 are shown in Table 4, whereas sensitivity, specificity, and discrimination accuracy are presented in Table 3. The methylation of cg07584066, cg16579770, and cg07763047 demonstrated also in Group 2 a relatively high accuracy (≥ 68%).

**Table 4:** Participants demographics and characteristics in Group 2 (independent test samples). Data are shown as the mean ± standard deviation. *p < 0.05 compared to HC.

	HC	AOD
No. of subjects	11	11
Sex (Male/Female)	5/6	5/6
Age (years)	53.8 ± 9.1	53.5 ± 7.6
Age at onset (years)	–	30.3 ± 9.6
SIGH-D	1.8 ± 1.9	21.3 ± 7.6*
Equivalent dose of imipramine (mg)	–	155 ± 91

AOD, adult-onset depression; HC, healthy controls; SIGH-D, Structured Interview Guide of the Hamilton Depression Rating Scale score.

Next, we assessed the discrimination performance in Group 2 using combinations of two cytosine methylation sites. We constructed the Bayes classifier using the training samples from Group 1b; the independent test samples from Group 2 were diagnosed using this Bayes classifier. The values for sensitivity, specificity, and discrimination accuracy of these combinations are shown in Table 5. The combination of cg16579770 and cg07763047 demonstrated with 73% a high accuracy. We further assessed the discrimination performance using combinations of three cytosine methylations (Table 5). The group consisting of cg07584066, cg16579770, and cg07763047 exhibited the highest accuracy (sensitivity, 64%; specificity, 91%; accuracy, 77%). Furthermore, we visualized the discrimination results for the test samples with the selected probe set, i.e., the target marker set (Supplementary Fig. S8a, b). The axes ${d}_{1}\left(\mathrm{x}\right)\mathrm{ and} {d}_{2}\left(\mathrm{x}\right)$, which are described in detail in the “Statistical analysis” paragraph of our Methods section, are the distances between a test sample x from Group 2 and the distribution of the HC and AOD samples from Group 1b, respectively. Our data show that the triple combination of cg07584066, cg16579770, and cg07763047 discriminates better than the dual combination of cg16579770 and cg07763047.

**Table 5:** Sensitivity, specificity, and accuracy using combinations of two or three DNA methylation sites for discrimination between AOD and HC.

	Sensitivity of test samples	Specificity of test samples	Accuracy of test samples
cg16579770, cg07763047	0.64	0.82	0.73
cg16579770, cg20903900	0.45	0.73	0.59
cg16579770, cg10294474	0.64	0.45	0.55
cg07763047, cg20903900	0.27	0.82	0.55
cg15971980, cg07584066	0.27	0.82	0.55
cg20903900, cg07584066	0.18	0.91	0.55
cg16579770, cg07584066, cg07763047	0.64	0.91	0.77
cg16579770, cg15971980, cg07584066	0.64	0.73	0.68
cg16579770, cg07763047, cg20903900	0.36	0.91	0.64
cg16579770, cg10294474, cg07584066	0.45	0.82	0.64
cg16579770, cg20903900, cg07584066	0.55	0.73	0.64

AOD, adult-onset depression; HC, healthy controls.

To confirm that the spread of the AOD distribution is smaller than that of the LOD distribution, the values of the determinant were calculated for both classes using cg16579770 and cg07763047 as specific AOD markers. The value of the determinant signifies the spread of the corresponding distribution. The ratio of AOD to HC was 0.066, whereas the ratio of LOD to HC was 3.187 (both p < 0.001; Supplementary Fig. S9). These results suggest that AOD was more homogeneous than HC, but LOD was less homogenous than HC.

## Discussion

Under the assumption that AOD is more strongly influenced by DNA methylation than LOD, we searched for methylation markers in AOD patients using a genome-wide DNA methylation array. As expected, the methylation levels of specific sites discriminated better between AOD and HC than between LOD and HC. Besides, we showed that the spread of the distribution in AOD is smaller than that in LOD.

The identification of specific DNA methylations may facilitate subtyping mental disorders. For example, several DNA methylation sites have been implicated in an increased risk of suicidal behavior in patients with bipolar disorder^16^. The DNA methylation of *FKBP5* was reported to be a potential indicator for the responsiveness to treatment by mindfulness-based stress reduction in post-traumatic stress disorder^17^. To our knowledge, the current study is the first demonstrating that the methylation profiles differ between AOD and LOD. Our results suggest that the vulnerability for AOD may be attributed to DNA methylation. LOD is associated with other illnesses, such as menopausal syndrome or cerebrovascular diseases rather than genetic risk^18^. Thus, DNA methylation markers may be suitable to diagnose AOD rather than LOD.

Several studies have examined genome-wide DNA methylation in the peripheral blood^19–28^. However, most of these studies were designed for symptomatic depression, did not validate their methylation markers by other methods such as pyrosequencing, or did not assess the accuracy using independent subjects. We included only patients who were diagnosed with MDD by psychiatrists, and we re-assessed six identified AOD methylation markers by pyrosequencing or amplicon bisulfite sequencing. Two methylated cytosines were validated by amplicon bisulfite sequencing, although the difference was small (< 5%) compared to HC. Furthermore, we validated the accuracy of these markers using independent subjects. In particular, the combination of cg07584066, cg16579770, and cg07763047 showed the highest accuracy. The locus cg07763047 is annotated to the gamma-aminobutyric acid (GABA) type A receptor β2 subunit (*GABRB2*) gene, cg16579770 to dihydroorotate dehydrogenase (*DHODH*), and cg07584066 to DEAH-box helicase 40 (*DHX40*).

*GABRB2* has been associated with schizophrenia^29,30^ and bipolar disorder^31^. One study in postmortem brains of elderly patients with depression reported that the *GABRB2* expression is significantly decreased in the anterior cingulate cortex in both MDD and bipolar disorder^32^. The GABA and glutamate neurotransmitter systems are also implicated in MDD and suicidal behavior^33^. *GABRB2* transcription is regulated by epigenetic mechanisms including methylation^32,34^. Our findings support a role for *GABRB2* in AOD.

The enzyme DHODH is involved in pyrimidine biosynthesis^35^. Pyrimidine biosynthesis pathways are connected to cell proliferation and metabolism, therefore DHODH is a focus for the development of new drugs against neoplastic or immunological disorders^36^. Until now, *DHODH* has not been known to be directly associated with MDD. However, DHODH and its pathways play a fundamental role in cellular homeostasis. For example, DHODH is essential for T cell proliferation, and inhibition of DHODH is beneficial in autoimmune diseases^37^. It has been suggested that inflammation contributes to the pathophysiology of depression^38^. A recent genome-wide methylation study showed that DNA methylation is altered at multiple immune-related loci in individuals with a history of depression^19^. Thus, *DHODH* may be associated with depression via neuroinflammation.

The protein DHX40 is an ATP-dependent DExD/H RNA helicase that exerts essential roles in RNA metabolism^39^. Unfortunately, these roles have not been well established yet; however, DExD/H RNA helicases are known to regulate ribosome biogenesis^40^. A recent blood transcriptome study reports that ribosomal genes are upregulated in stress^41^. Thus, ribosomal biogenesis may contribute to AOD pathophysiology.

A recent meta-analysis of epigenome-wide studies in middle-aged and elderly subjects associated the methylation of three CpG sites with depressive symptoms^12^. Unfortunately, we did not replicate these findings. In our study, we diagnosed MDD strictly and selected for the epigenome-wide analysis only MDD patients with an age of 50 years or older. By contrast, the meta-analysis consisted of nine population-based cohort studies including a cardiovascular study, most of the participants were not diagnosed with MDD, and their depressive symptoms were only assessed by self-reported questionnaires.

Several limitations of this study should be mentioned. First, the sample size was small compared to the number of analyzed CpG sites. It is likely that some false positive markers were detected or some false negative markers remained undetected. Second, we only examined methylation markers using leukocytic DNA based on future applicability for clinical use. The functions in the brain of the annotated genes and their methylation remain unclear. Third, most patients received antidepressants. We cannot exclude the possibility that antidepressants might have altered the methylation levels of candidate CpG sites. Fourth, clinical parameters might have influenced the methylation levels. A few factors were indicated to influence the AOD and HC classification by scatter plots representing the relationships between age, age of onset, number of depressive episodes, Structured Interview Guide of the Hamilton Depression Rating Scale (SIGH-D) score, white blood cell (WBC) count, ratio of neutrophils, and methylation levels. Specifically, the methylation levels of the cg07763047 and cg10294474 sites correlated with age at onset, suggesting that the detected markers were onset-age-dependent. However, other clinical factors might have influenced the methylation status. Finally, we did not validate that the methylation markers can distinguish AOD from other psychiatric diseases including schizophrenia and bipolar disorder.

In summary, we detected several methylation markers to discriminate AOD from HC populations with high accuracy. The alteration of DNA methylation in AOD differed from that in LOD. The spread of the distribution in AOD was smaller than those in HC and LOD. These results suggest that methylation markers of MDD should be examined under specific consideration of the onset age.

## Materials and methods

### Subjects

The Institutional Review Boards of Yamaguchi University Hospital and Gunma University Hospital approved this study, and all subjects were fully informed about study aims and procedures before providing written informed consent for participation. This study was carried out in accordance with the latest version of the Declaration of Helsinki.

### Participants in Group 1

All participants were recruited between 2012 and 2013. Elderly (age ≥ 50 years) patients with MDD and healthy participants were enrolled as previously described^8^. Briefly, patients with MDD, as well as HC participants, were assessed using clinical interviews, as well as a structured interview of the International Neuropsychiatric Interview (M.I.N.I., Japanese version 5.0.0), which is structured in accord with the DSM-IV^42^. We also defined a depressed state by a score of more than 18 using the SIGH-D^43^. Patients in remission met the DSM-IV criteria for full remission. Exclusion criteria for MDD patients included current or history of substance abuse/dependence, other psychiatric illnesses, other medical conditions (e.g., neurological diseases, severe liver failure, unstable hypertension), or family history of hereditary neurological disorders.

The samples of AOD participants (age of onset < 50 years) in a depressed state (N = 10), LOD participants (onset age ≥ 50 years) in a depressed state (N = 25), and HC participants (N = 30) were analyzed by genome-wide DNA methylation assays. Their demographic data are summarized in Table 1a (Group 1a; discovery samples). The AOD participants (N = 10), a part of the LOD group (N = 10), and some HC participants (N = 10) were additionally evaluated using amplicon bisulfite sequencing and pyrosequencing. Their demographic data are summarized in Table 1b (Group 1b; training samples). In Group 1a, the following values were missing: the total duration parameter in the LOD group (1); the number of episodes in the AOD group (1); and the percentages of each WBC type in the HC (4), AOD (1), and LOD (2) groups. In contrast, the following values were missing in Group 1b: the number of episodes in the AOD group (1) and the percentages of each WBC type in the HC (1) and AOD (1) groups.

### Participants in Group 2

The second group consisted of 11 AOD and 11 HC participants at the Yamaguchi site and the Gunma site. We enrolled 5 patients with AOD and 5 HC participants at the Yamaguchi site, as well as 6 AOD and 6 HC subjects at the Gunma site^8,44^, and the samples were collected between 2012 and 2015. All AOD patients and HC participants met the inclusion and exclusion criteria according to the protocol described for Group 1 with the only differences being the age restriction (age ≥ 20 years) and the SIGH-D scores (≥ 14). The demographic data are summarized in Table 4 (Group 2; independent test sample).

### Genome-wide DNA methylation array

Venous blood samples were collected from all participants between 9:00 am and 12:00 pm. Genomic DNA was purified from peripheral blood cells using the QIAamp DNA Blood Midi Kit (Qiagen, Chatsworth, CA, USA) according to the manufacturer’s manual. The DNA quality was determined using a Spectrophotometer U-2900 (Hitachi High-Technologies, Tokyo, Japan), and an O.D. 260/280 ratio ≥ 1.5 was obligatory to consider samples for further analyses. DNA quantity was determined using the Quant-iT dsDNA Assay Kit, broad range (Thermo Fisher Scientific Inc., Waltham, MA, USA). Bisulfite conversion of 500 ng genomic DNA was performed using the EZ DNA Methylation Kit (Zymo Research, Irvine, CA, USA). The DNA methylation level was assessed according to the manufacturer’s protocols using Infinium HumanMethylation450 BeadChips (Illumina Inc., San Diego, CA, USA). DNA methylation data were analyzed with GenomeStudio (version 2011.1) and Methylation Module (version 1.9.0; both Illumina Inc.). The raw data were normalized using the beta-mixture quantile normalization method^45^. The methylation ratio of each CpG site was determined as a beta-value from 0 (completely unmethylated) to 1 (fully methylated). The probes for the analysis of methylation markers were excluded according to the following conditions: (1) the detection p-value was less than 0.01 in at least one sample, (2) the beta-value could not be calculated for at least one probe, and (3) all probes of single-nucleotide polymorphisms (rs#). The final data included 411,383 probes of CpG sites. The probes for the RefSeq analysis were excluded as follows: (1) the detection p-value was less than 0.01 in at least one sample and (2) all probes of single-nucleotide polymorphisms (rs#). These data finally included 481,247 probes. The RefSeq genes were identified according to the accession numbers of the probes. If multiple probes were associated with a promotor region, the median methylation frequency was calculated.

### Pyrosequencing

Pyrosequencing was performed using the standard methods cited in a previous study^46^. Polymerase chain reaction (PCR) and sequencing primers (see Supplementary Table S5) were designed using Pyrosequencing Assay Design Software v2.0 (Qiagen).

Genomic DNA (200 ng) was bisulfite-treated using the EZ DNA Methylation-Gold Kit (Zymo Research) according to the manufacturer’s protocol. Briefly, the reaction was performed 10 min at 98 °C and 2.5 h at 64 °C, then samples were stored at 4 °C. The converted samples were washed and desulfonated with M-wash and M-desulfonation buffers on a Zymo-Spin IC Column. The converted genomic DNA was eluted by adding 20 µl elution buffer. DNA samples were stored at -20 °C until further use.

PCRs were carried out in a volume of 20 µl with 20 ng or more of converted DNA, 2.5 µl 10 × Taq buffer, 5 units Hot Start Taq Polymerase (Qiagen), 2 µl 2.5 mM dNTP mixture, 1 µl 10 pmol/µl Primer-S, and 1 µl 10 pmol/µl biotinylated-Primer-As. The amplification was carried out according to the general guidelines suggested for pyrosequencing: denaturation at 95 °C for 10 min, followed by 45 cycles at 95 °C for 30 s, at 48 °C for 30 s, and at 72 °C for another 30 s, and a final extension at 72 °C for 5 min. The ssDNA template was prepared from 16 to 18 µl biotinylated PCR product using Streptavidin Sepharose High Performance beads (GE Healthcare, Chicago, IL, USA) following the PSQ 96 sample preparation guide. Gel images of the PCR products are shown in Supplementary Fig. S10. For the analysis, 15 pmol of the respective sequencing primer were added. Sequencing was performed using a PyroMark ID system with the PyroMark Gold Reagents kit (Qiagen) according to the manufacturer’s instruction without further optimization. The methylation percentage was calculated as the average of the degree of methylation at each CpG site formulated in pyrosequencing.

### Amplicon bisulfite sequencing

For validation of the whole-genome methylation array data, we selected the loci of seven identified genes. Each primer for amplicon bisulfite sequencing was designed using the Methyl Primer Express software v1.0 (Thermo Fisher Scientific Inc.). Sodium bisulfite conversions of 500 ng genomic DNA were performed using the MethylEasy Xceed Rapid DNA Bisulphite Modification Kit (Genetic Signatures, New South Wales, Australia). PCR amplification of bisulfite-treated DNA was performed using TAKARA EpiTaq HS (Takara Bio Inc., Kusatsu, Japan). The list of primer sequences and the summary of the PCR conditions are presented in Supplementary Table S6. The PCR products were directly cleaned up using NucleoSpin Gel (Takara Bio Inc.) and a PCR Clean-up kit (Macherey–Nagel GmbH & Co. KG, Düren, Germany). Gel images of the PCR products are shown in Supplementary Fig. S11. The sequence libraries were produced using the TruSeq Nano DNA LT Library Prep Kit (Illumina Inc.). The qualities of the libraries were checked using an Agilent 2100 BioAnalyzer (Agilent Technologies Inc., Santa Clara, CA, USA). Sequencing of 250 bp paired-end reads was generated using the Illumina MiSeq platform with MiSeq Reagent Kit ver3 (Illumina Inc.). Sequence data were analyzed using Genedata Expressionist for Genomic Profiling ver9.1.4a (Genedata Inc., Basel, Switzerland). Each read was trimmed to 150 bp, and mapping was performed relative to the h19/GRCh37 assembly of the human genome using Bismark Bisulfite Mapper ver0.14.4^47^. Percentages of methylcytosines were calculated using Bismark Bisulfite Mapper ver0.14.4.

### Statistical analysis

Age distributions, total durations of all MDD episodes, and numbers of episodes of the participants were evaluated using the unpaired Student’s t-test or the one-way analysis of variance (ANOVA) with the Tukey's post hoc test, whereas the sex distribution was analyzed by Fisher's exact test using the EZR software (version 1.37)^48^. The number of WBCs and the percentage of each WBC type were evaluated using the one-way ANOVA with the Tukey's post hoc test. Missing values were deleted using pairwise deletion methods.

We selected candidate methylation markers from 411,383 probes of CpG sites using the F-ratio as previously reported^49^. The F-ratio has been used as a popular criterion in feature selection of statistical pattern recognition. The F-ratio, ${f}_{j}$, for probe, $j$, is defined by:

$${f}_{j}=\frac{{({\mu }_{1j}-{\mu }_{2j})}^{2}}{{\sigma }_{1j}^{2}+{\sigma }_{2j}^{2}}$$

where ${\mu }_{ij}$ and ${\sigma }_{ij}^{2}$ are the sample mean and sample variance, respectively, estimated from probe, $j$, using training samples from class, $i (i=\mathrm{1,2})$. The F-ratio measures the difference between two sample means normalized by the averaged sample variances. In feature selection, the F-ratio for each probe is first computed and probes are then ranked in order of decreasing F-ratio magnitude. The best probe set is given by some of the highest F-ratios.

The normal distribution of the methylation array data was checked using the Kolmogorov–Smirnov test. Pearson’s correlation coefficients were used to measure correlations between participant age, onset age, number of episodes, total duration of all MDD episodes, SIGH-D scores, number of WBCs, percentages of each WBC type, and methylation level of each site. A false discovery rate method (Benjamini–Hochberg adjustment) was used to correct p-values.

A ROC analysis was carried out using EZR. We determined the optimal cut-off points by maximizing the Youden index (sensitivity + specificity − 1)^50^.

The discrimination performance was assessed as follows: two or three target markers were selected from the six identified probes (cg15794987, cg07763047, cg21347377, cg17458347, cg14567489, and cg16579770). Using the methylation levels of pyrosequencing or amplicon bisulfite sequencing as distinguishing features, we had a two- or three-dimensional vector x, whose components were the methylation levels. First, the sample mean vector ${\mu }_{i}$ and the sample covariance matrix ${\sum }_{i}$ of class ${\omega }_{i}$ were estimated using 10 training samples from AOD and HC participants in Group 1b. The resulting Bayes classifier was assigned to a test sample x being classified to class ${\omega }_{1}$, i.e., HC, if

$${d}_{1}\left(x\right)<{d}_{2}\left(x\right)$$

where $${d}_{i}=\frac{1}{2}{(x-{\mu }_{i})}^{T}{\sum }_{i}^{-1}\left(x-{\mu }_{i}\right), i=1, 2$$.

For the methylation distribution analysis, we calculated the values of determinants using the methylation levels of the probes cg16579770 and cg07763047. In these calculations, 10 AOD samples were fixed, and 10 samples were 100 times randomly extracted from either the 30 HC samples or 25 LOD samples to calculate the average determinant of HC or LOD. The one-tailed t-test was performed with the null hypothesis that the ratio to HC is 1.

A summary of the statistical strategy used in this study is shown in Supplementary Fig. S12.

## Supplementary Information

Supplementary Information 1.

Supplementary Information 2.

## Data Availability

All data in this published article (and its Supplementary Information files) are available. The other data analyzed in the current study are not publicly available for ethical reasons.
