# Supplementary material for: Distinct epigenetic signatures between adult-onset and late-onset depression
Source: Sci Rep. 2021 Jan 27;11:2296. doi: 10.1038/s41598-021-81758-8 (PMC7840753; doi:10.1038/s41598-021-81758-8)

Distinct epigenetic signatures between adult-onset and late-onset depression

Hirotaka Yamagata[1,2]*, Hiroyuki Ogihara[3], Koji Matsuo[1,4], Shusaku Uchida[1,2,5], Ayumi Kobayashi[1], Tomoe Seki[1,2], Masaaki Kobayashi[1], Kenichiro Harada[1], Chong Chen[1], Shigeo Miyata[6,7], Masato Fukuda[7], Masahiko Mikuni[7,8,9], Yoshihiko Hamamoto[3], Yoshifumi Watanabe[1,10] & Shin Nakagawa[1]

1 Division of Neuropsychiatry, Department of Neuroscience, Yamaguchi University Graduate School of Medicine, 1-1-1 Minami-kogushi, Ube, Yamaguchi 755-8505, Japan

2 Core Research for Evolutional Science and Technology (CREST), Japan Science and Technology Agency (JST), 4-1-8 Honcho, Kawaguchi, Saitama 332-0012, Japan

3 Division of Electrical, Electronic and Information Engineering, Graduate School of Sciences and Technology for Innovation, Yamaguchi University, 2-16-1 Tokiwadai, Ube, Yamaguchi 755-8611, Japan

4 Present address: Department of Psychiatry, Faculty of Medicine, Saitama Medical University, 38 Morohongo, Moroyama, Iruma, Saitama 350-0495, Japan

5 Present address: SK project, Medical Innovation Center, Kyoto University Graduate School of Medicine, 53 Shogoin-Kawahara-cho, Sakyo-ku, Kyoto 606-8507, Japan

6 Department of Genetic and Behavioral Neuroscience, Gunma University Graduate School of Medicine, 3-39-22 Showa-machi, Maebashi, Gunma 371-8511, Japan

7 Department of Psychiatry and Neuroscience, Gunma University Graduate School of Medicine, 3-39-22 Showa-machi, Maebashi, Gunma 371-8511, Japan

8 Present address: Department of Psychiatry, Hokkaido University, Graduate School of Medicine, Kita 15, Nishi 7, Kita-ku, Sapporo 060-8638, Japan

9 Hakodate Watanabe Hospital, 1-31-1 Yunokawacho, Hakodate, Hokkaido 042-8678, Japan

10 Present address: Southern TOHOKU Research Institute for Neuroscience, Southern TOHOKU General Hospital, 7-115 Yatsuyamada, Koriyama, Fukushima 963-8052, Japan

*Correspondence to:

Hirotaka Yamagata, M.D., Ph.D.

Division of Neuropsychiatry, Department of Neuroscience, Yamaguchi University Graduate School of Medicine, 1-1-1 Minami-kogushi, Ube, Yamaguchi 755-8505, Japan

TEL: +81 836 22 2255; FAX: +81 836 22 2253

E-mail: gata@yamaguchi-u.ac.jp

Supplementary information

Supplementary Fig. S1 Scatter plots between the array data and the pyrosequencing or the amplicon bisulfite sequencing data using the EZR software (http://www.jichi.ac.jp/saitama-sct/SaitamaHP.files/statmedEN.html).

Supplementary Fig. S2 Scatter plots between age and the methylation levels of each site.

Supplementary Fig. S3 Scatter plots between age at onset and the methylation levels of each site.

Supplementary Fig. S4 Scatter plots between number of episodes and the methylation levels of each site.

Supplementary Fig. S5 Scatter plots between WBCs and the methylation levels of each site.

Supplementary Fig. S6 Scatter plots between neutrophils (%) and the methylation levels of each site.

Supplementary Fig. S7 Scatter plots between SIGH-D scores and the methylation levels of each site.

Supplementary Fig. S8 Discrimination performance using combinations of two cytosine methylation sites. Comparison of the discrimination ability using two (cg16579770 and cg07763047) (**a**) or three (cg07584066, cg16579770, and cg07763047) (**b**) DNA methylation sites.

Supplementary Fig. S9 Comparison of the spread among AOD, LOD, and HC distributions using cg16579770 and cg07763047.

The ratio of the determinants of AOD or LOD to HC are shown. The statistical test between cg16579770 and cg07763047 for discriminating AOD from HC.

Supplementary Fig. S10 Gel images of PCR products for pyroseqencing. Five micro-litters of DNA Ladder 1kb plus (BioFact) were used for loading controls and markers.

Supplementary Fig. S11 Gel images of PCR products for amplicon bisulfite sequencing. Gel imaging was performed ATTO printgraph and ATTO imageSaver AE-6905C. Five micro-litters of GeneRuler 100 bp Plus DNA Ladder (Thermo Fisher Scientific) were used for loading controls and markers.

Supplementary Fig. S12 A summary of statistical strategy.

Supplementary Table S1 The 100 top-ranked DNA methylation sites in AOD and LOD samples.

Supplementary Table S2 The 100 top-ranked RefSeq genes in AOD and LOD samples.

Supplementary Table S3 Validation of specific DNA methylation sites.

(**a**) Correlation between the methylation levels in pyrosequencing and the genome-wide DNA methylation array. (**b**) Correlation between the methylation levels in amplicon bisulfite sequencing and the genome-wide DNA methylation array. Q-values were calculated using the false discovery rate method (the Benjamini-Hochberg adjustment method).

Supplementary Table S4 Correlation between the methylation levels and each parameter.

Q-values were calculated using the false discovery rate method (the Benjamini-Hochberg adjustment method).

Supplementary Table S5 Primer list for pyrosequencing. PCR and sequencing primers were designed using Pyrosequencing Assay Design Software v2.0 (Qiagen).

Supplementary Table S6 Primer list and PCR conditions for amplicon sequencing. Each primer was designed using the Methyl Primer Express software v1.0 (Thermo Fisher Scientific Inc.).

Figure S1 Scatter plots between the array data and the pyrosequencing or the amplicon bisulfite sequencing data

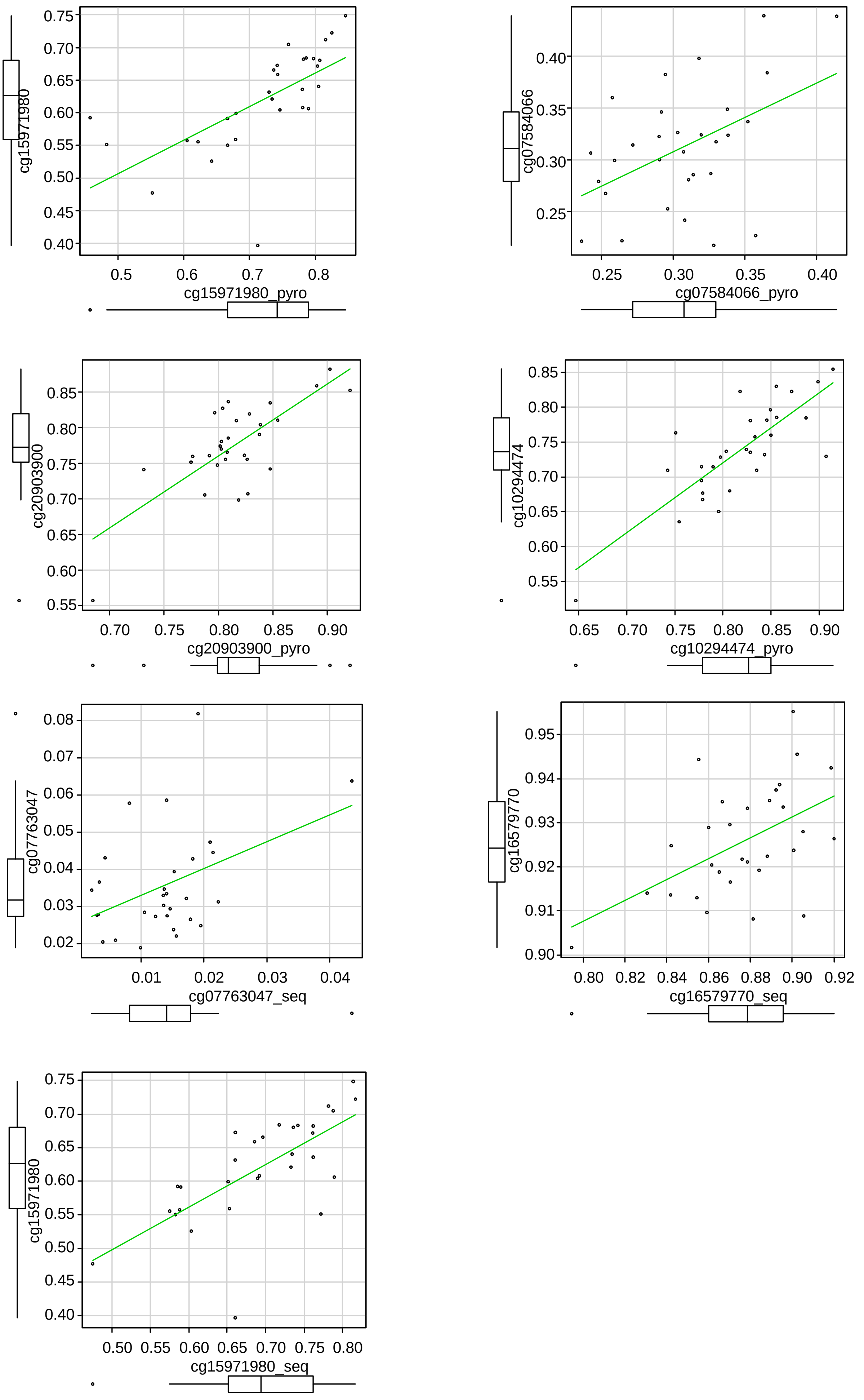

Figure S2 Scatter plots between age and the methylation levels of each site

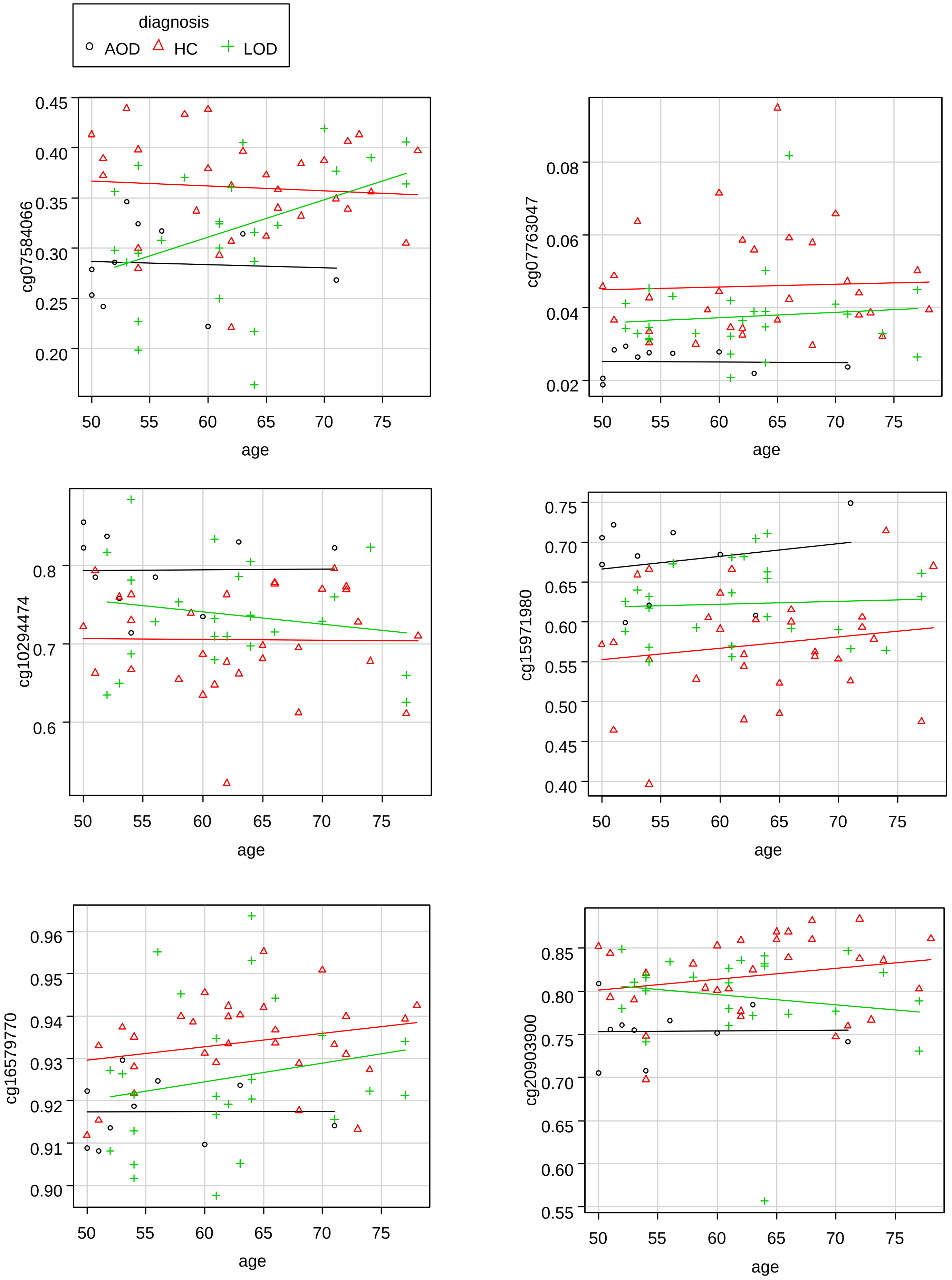

Figure S3 Scatter plots between age at onset and the methylation levels of each site

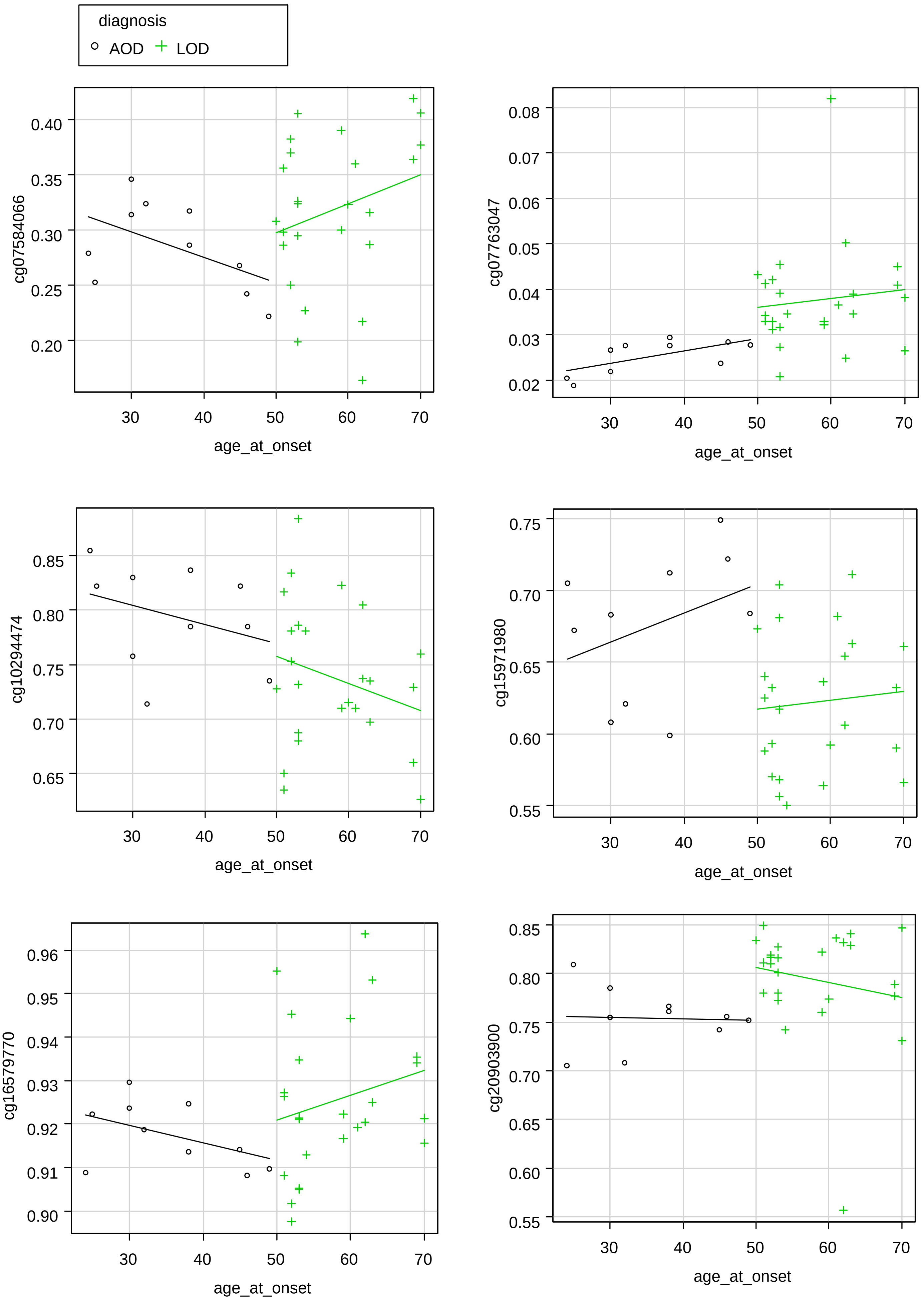

Figure S4 Scatter plots between number of episodes and the methylation levels of each site

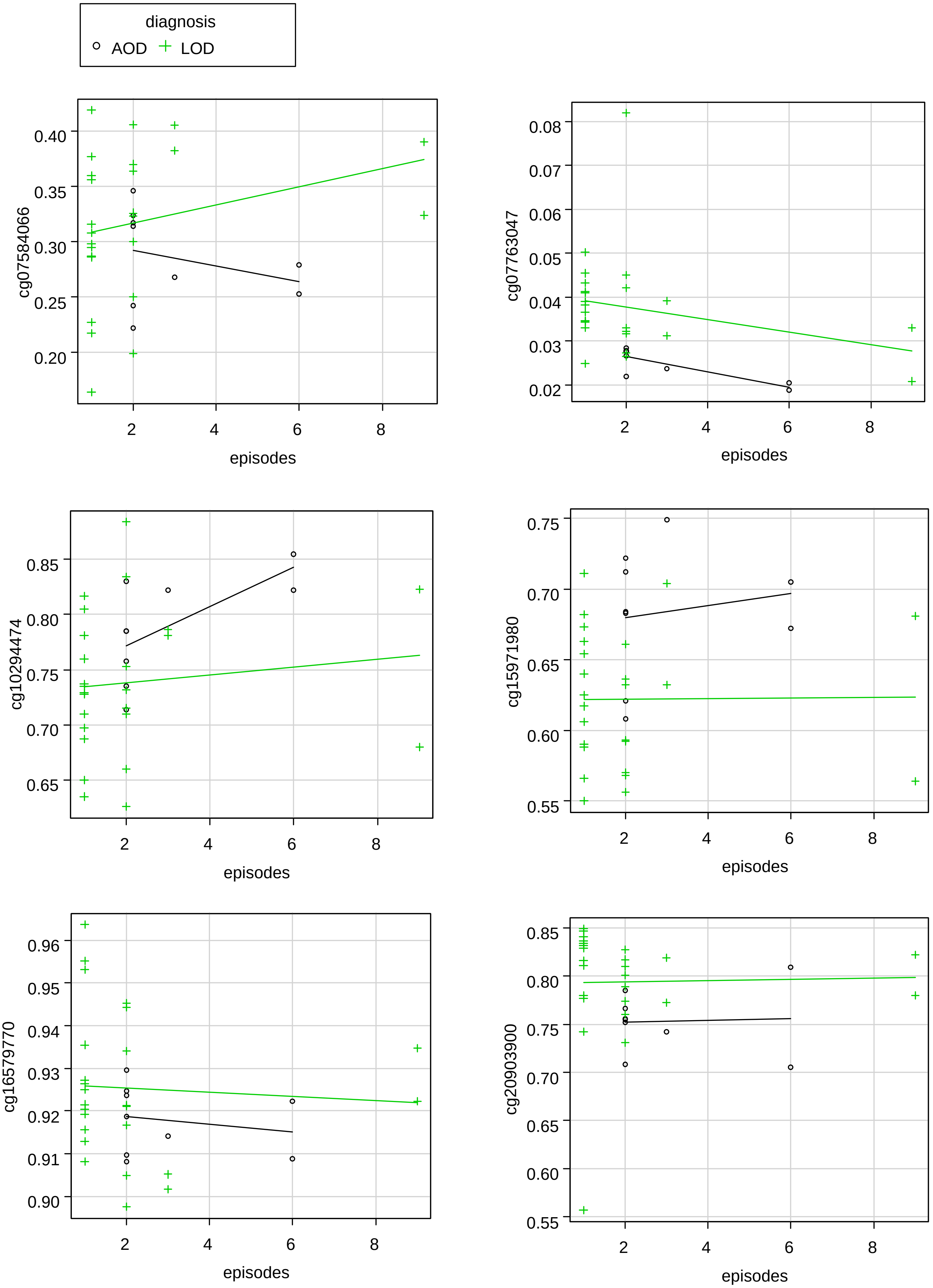

Figure S5 Scatter plots between WBCs and the methylation levels of each site

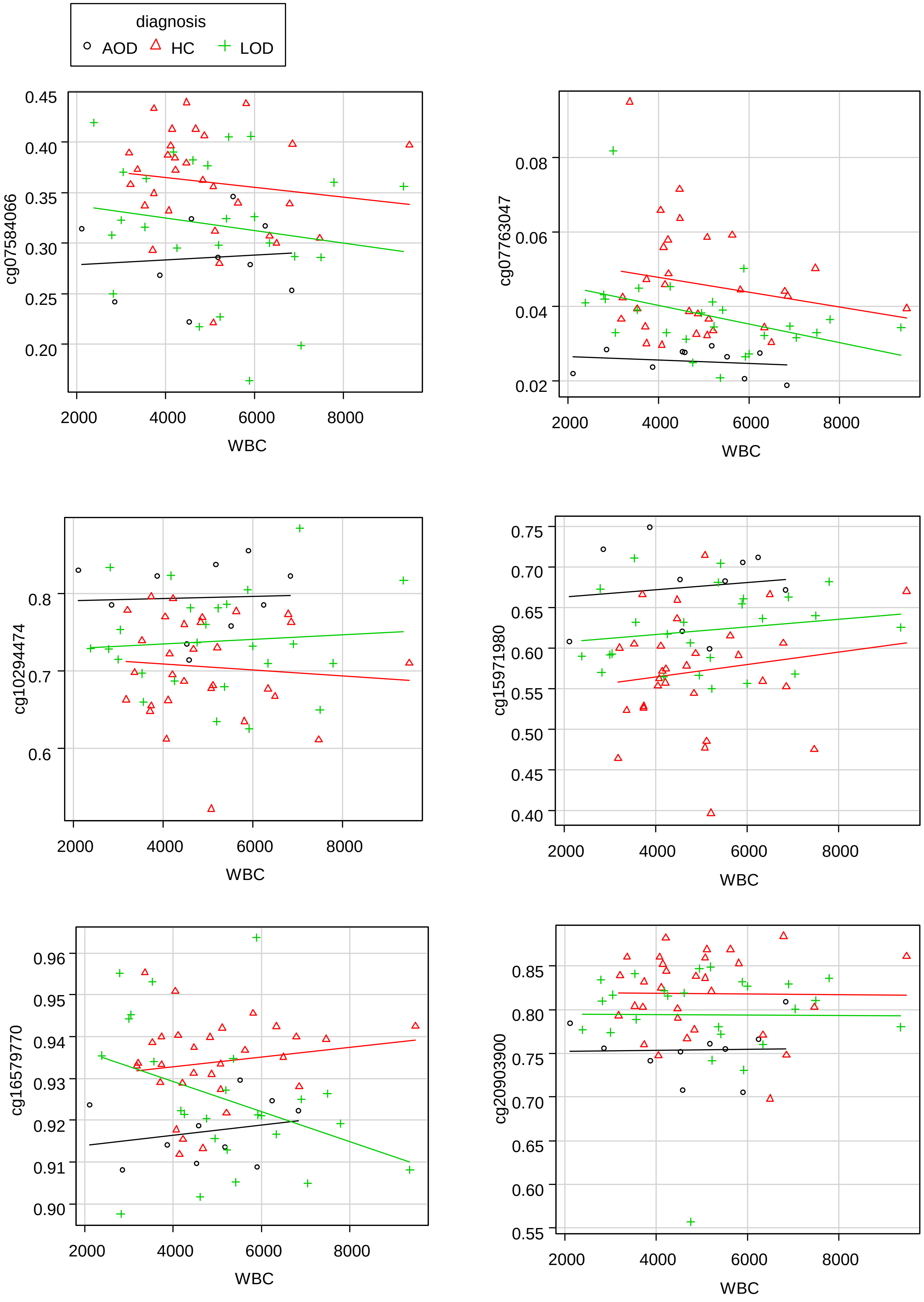

Figure S6 Scatter plots between neutrophils (%) and the methylation levels of each site

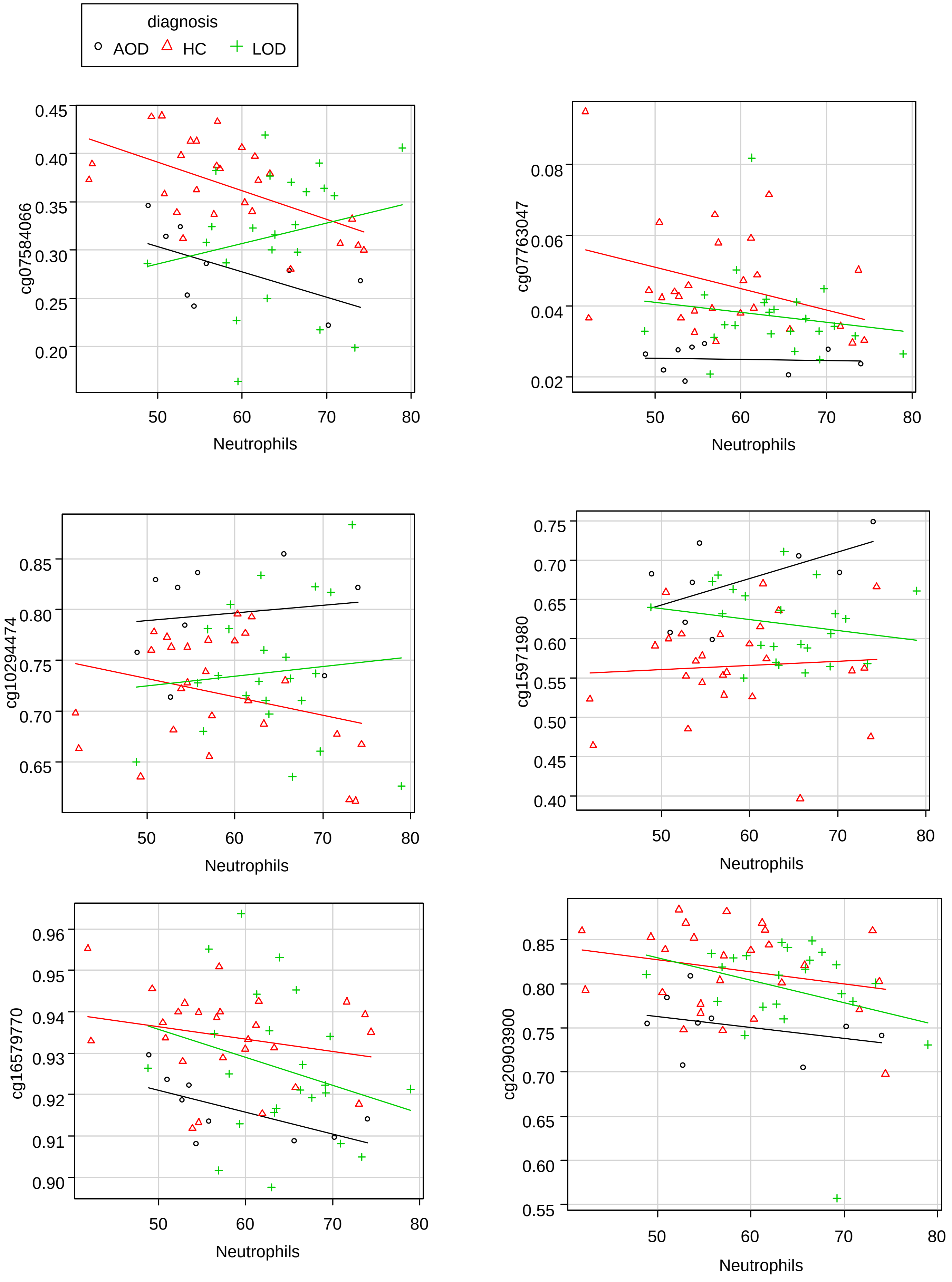

Figure S7 Scatter plots between SIGH-D scores and the methylation levels of each site

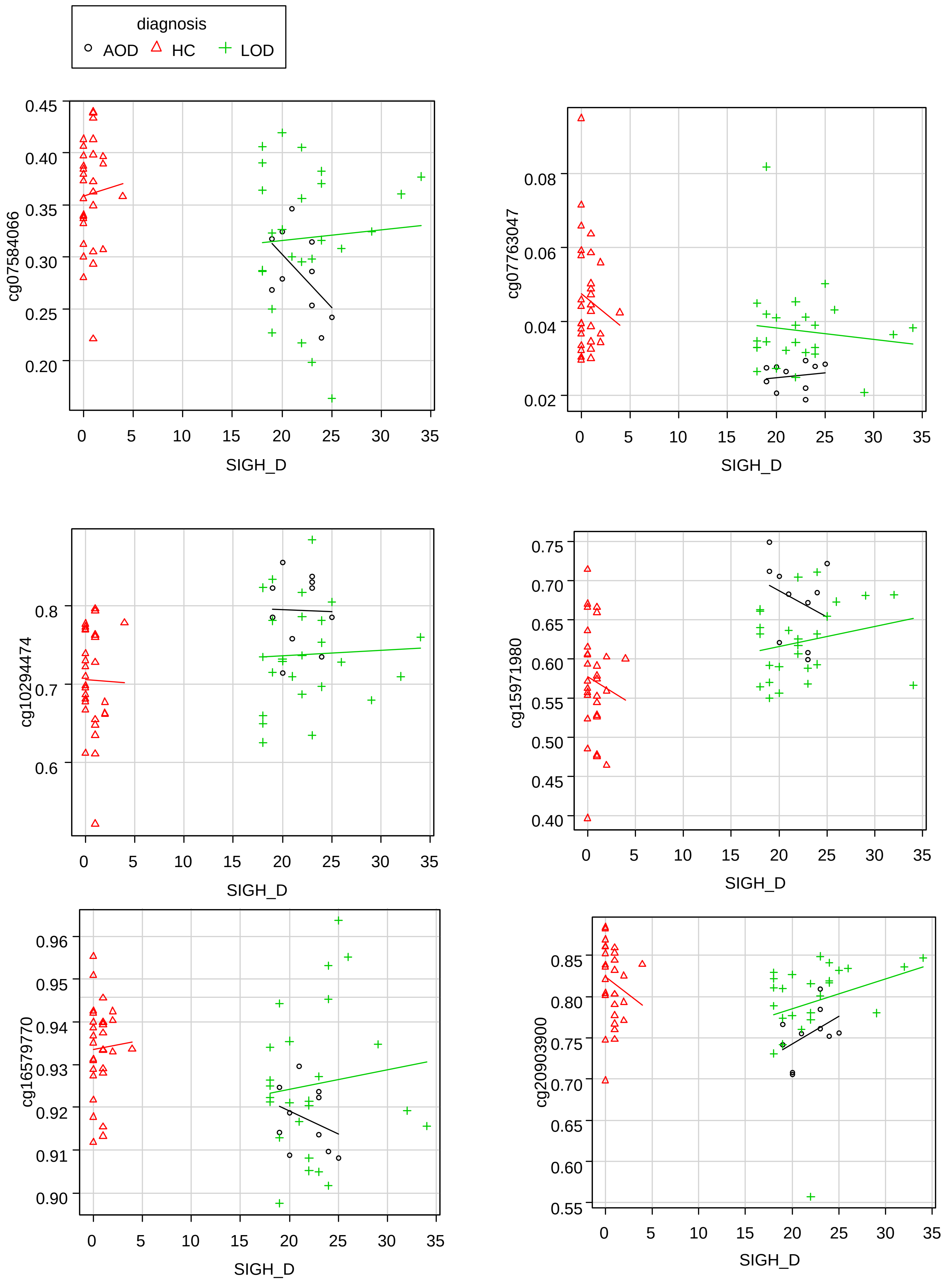

Figure S8a

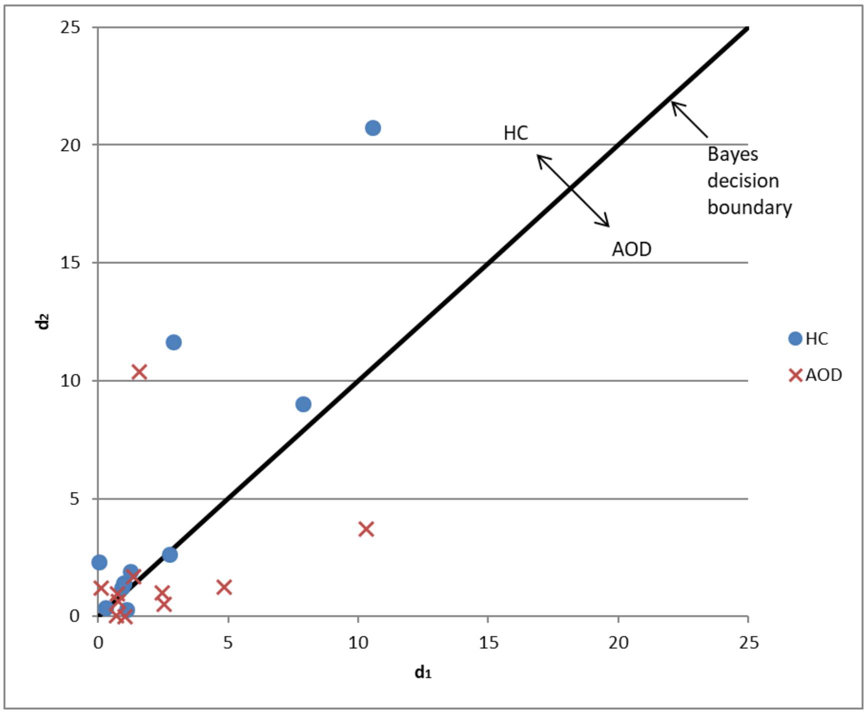

Use of cg16579770 and cg07763047

Figure S8b

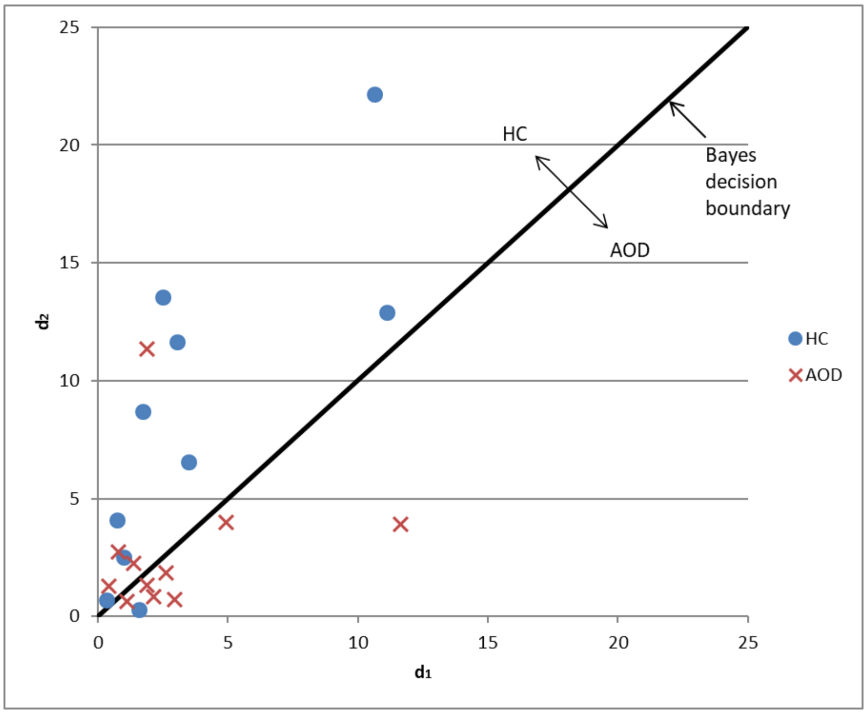

Use of cg07584066, cg16579770 and cg07763047

Supplementary Fig. S8 Discrimination performance using combinations of two cytosine methylation sites

Comparison of the discrimination ability using two (cg16579770 and cg07763047) (a) or three (cg07584066, cg16579770, and cg07763047) (b) DNA methylation sites

Figure S9

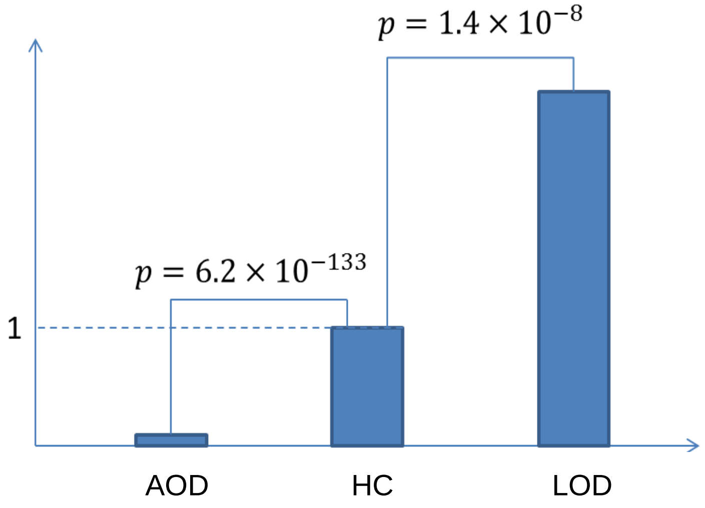

Supplementary Fig. S9 Comparison of the spread among AOD, LOD, and HC distributions using cg16579770 and cg07763047.
The ratio of the determinants of AOD or LOD to HC are shown. The statistical test between cg16579770 and cg07763047 for discriminating AOD from HC.

Figure S10

Gel images of PCR products

Samples of pyrosequencing

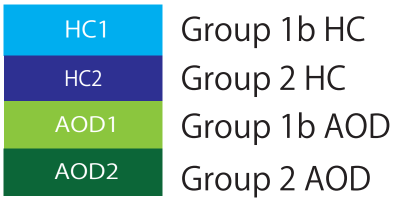

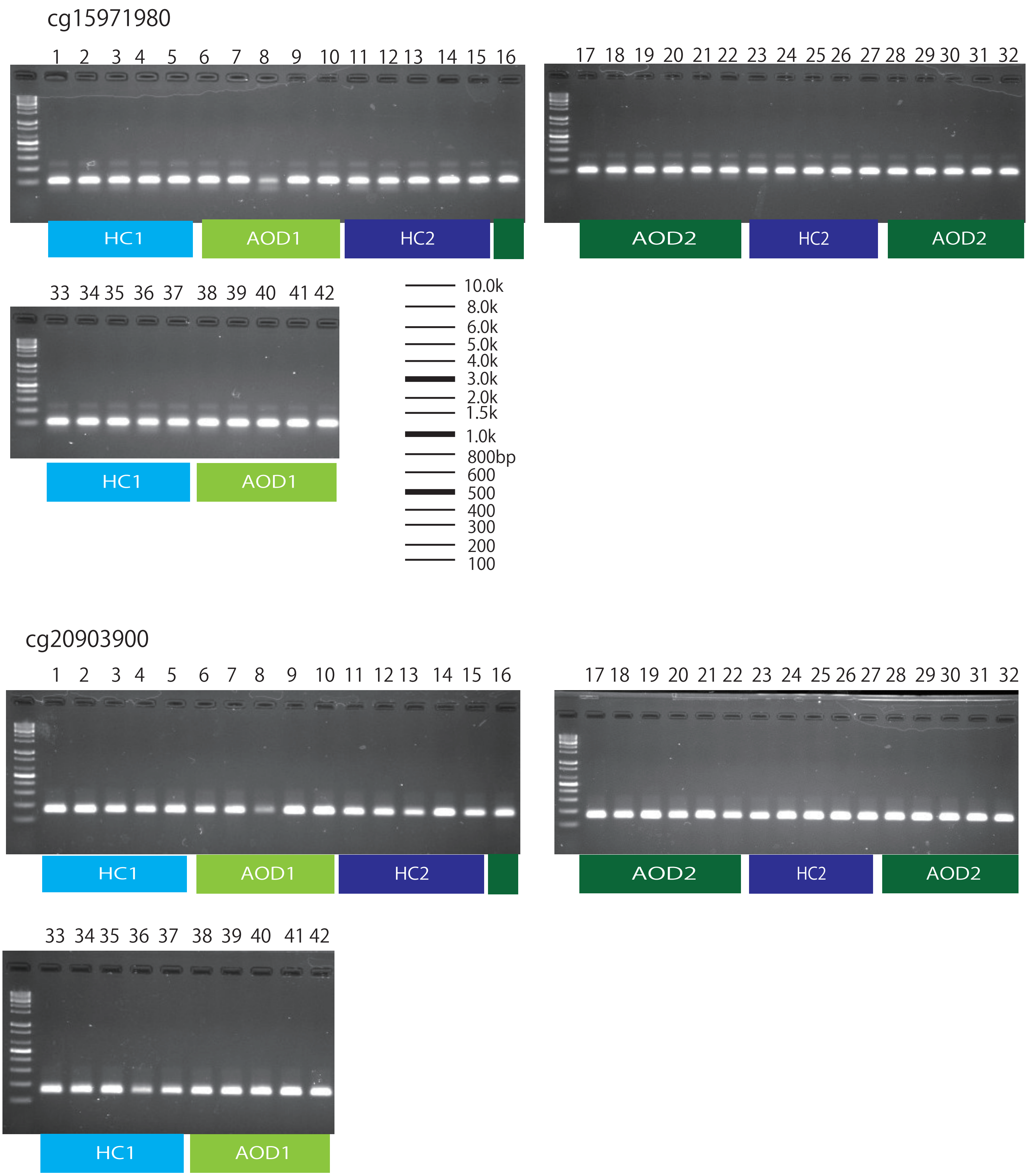

cg10294474

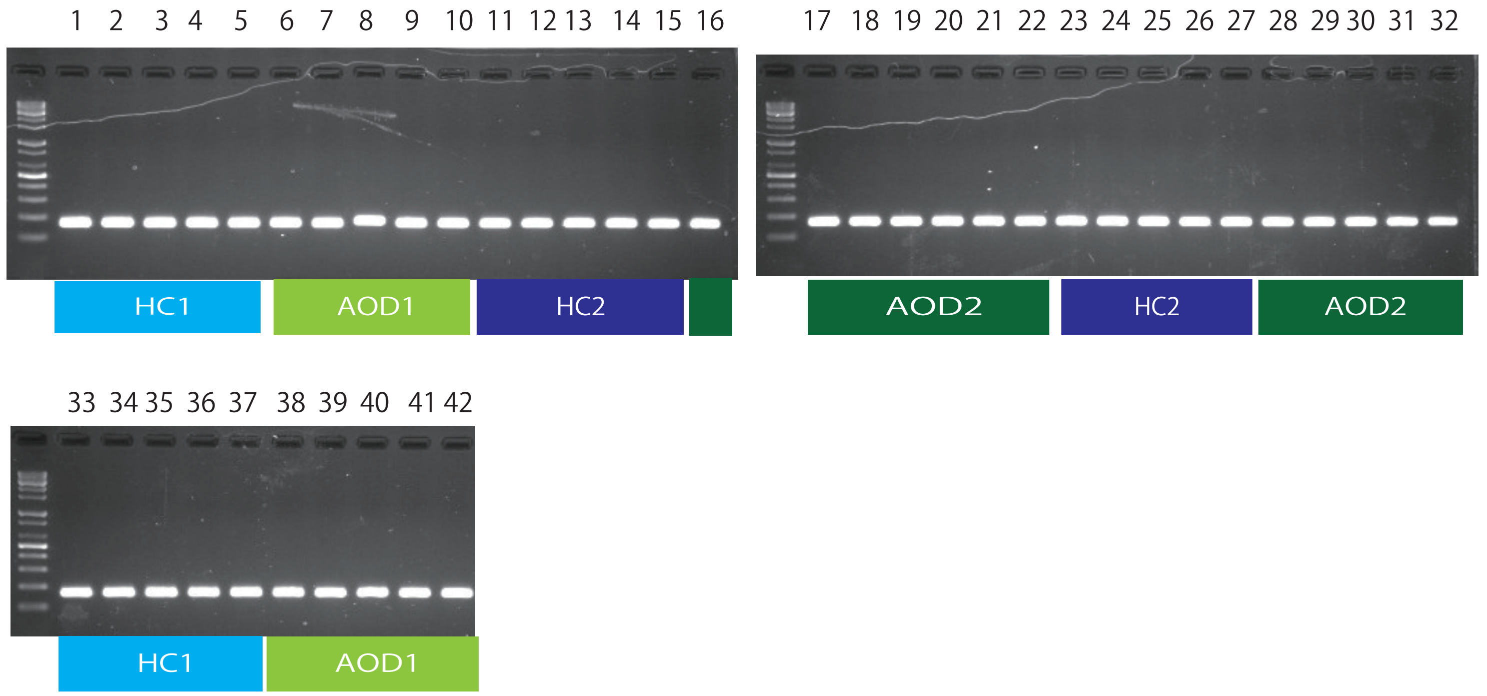

cg07584066

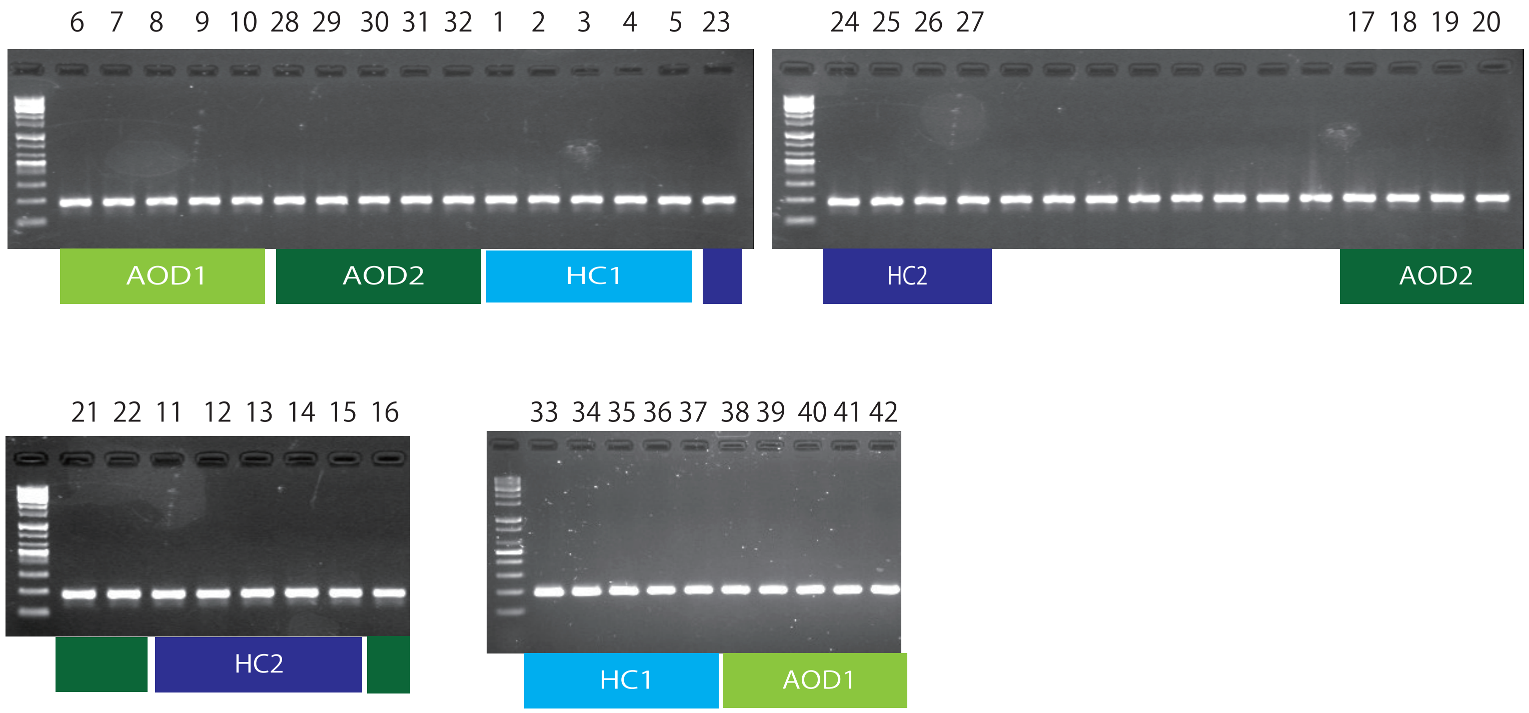

Figure S11

Gel images of PCR products

Samples of amplicon bisulfite sequencing

HC1 Group 1b HC
HC2 Group 2 HC
AOD1 Group 1b AOD
AOD2 Group 2 AOD

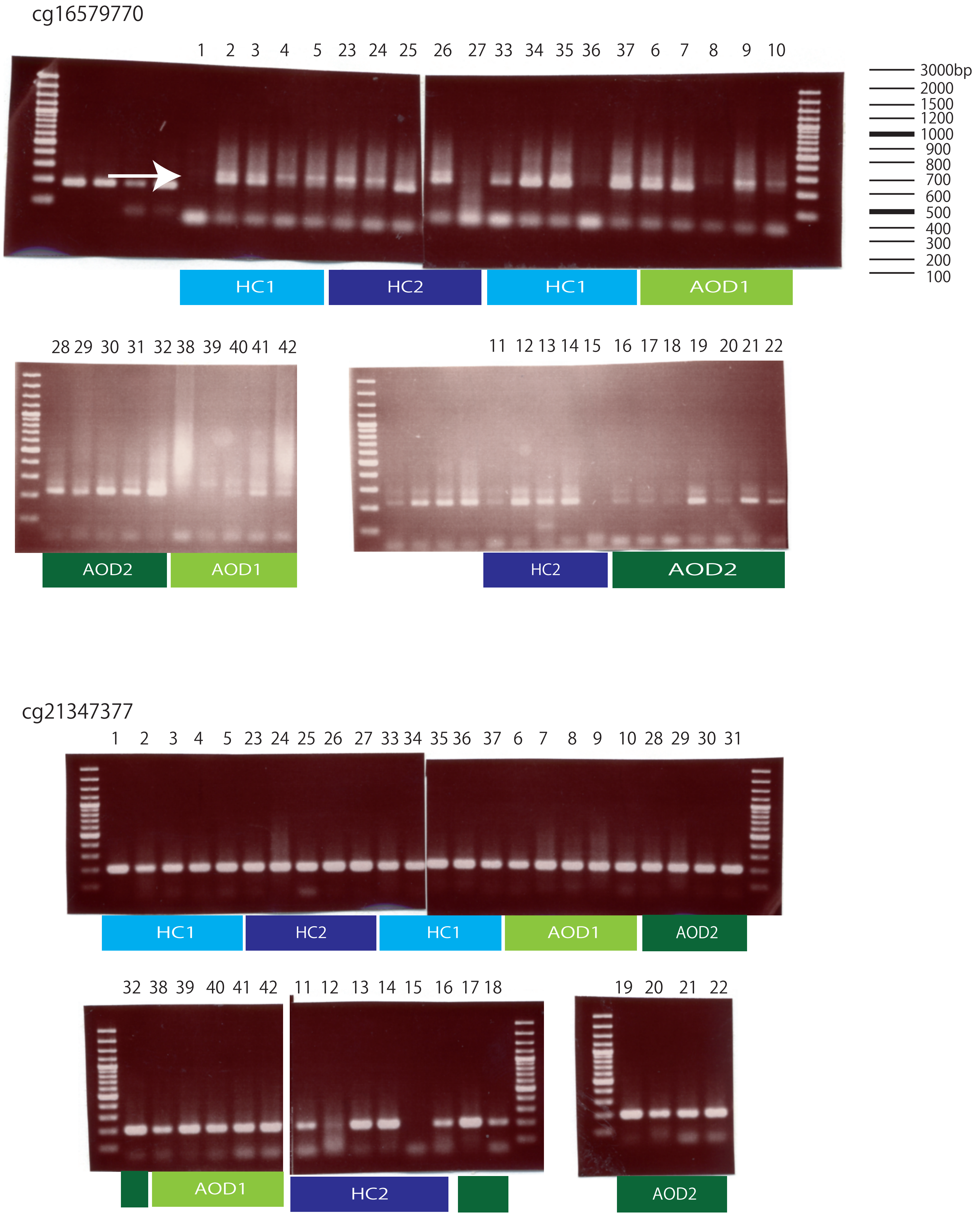

cg07763047

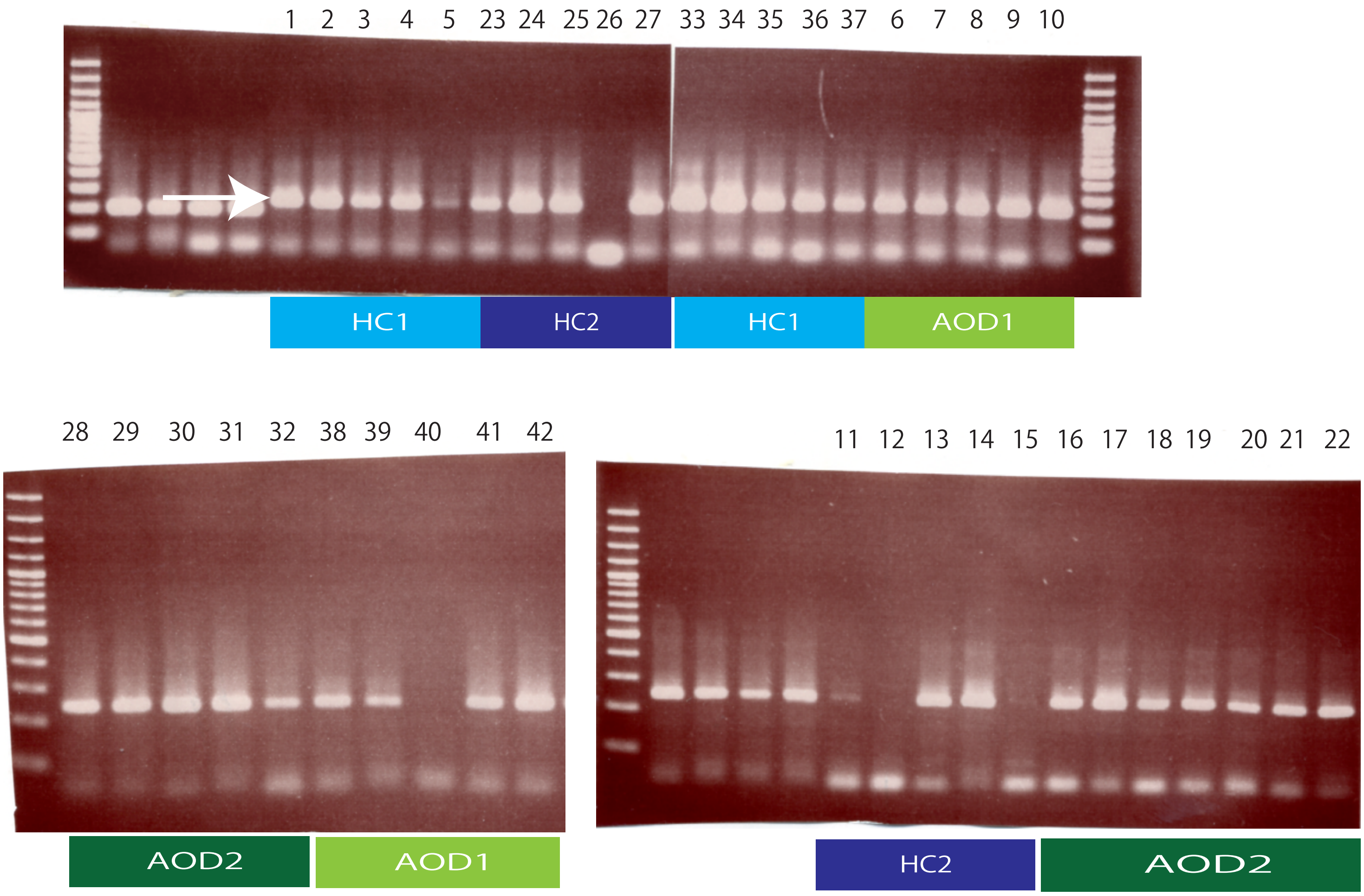

cg14567489

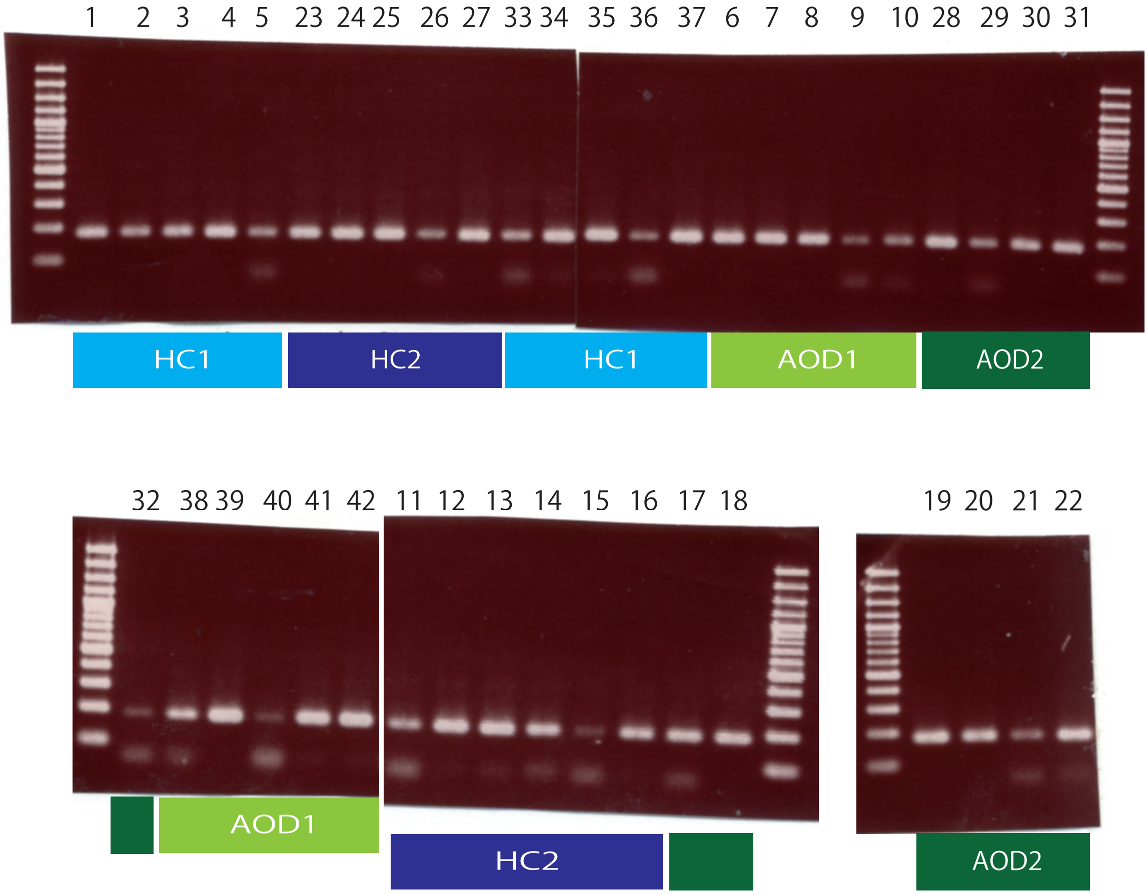

cg17458347

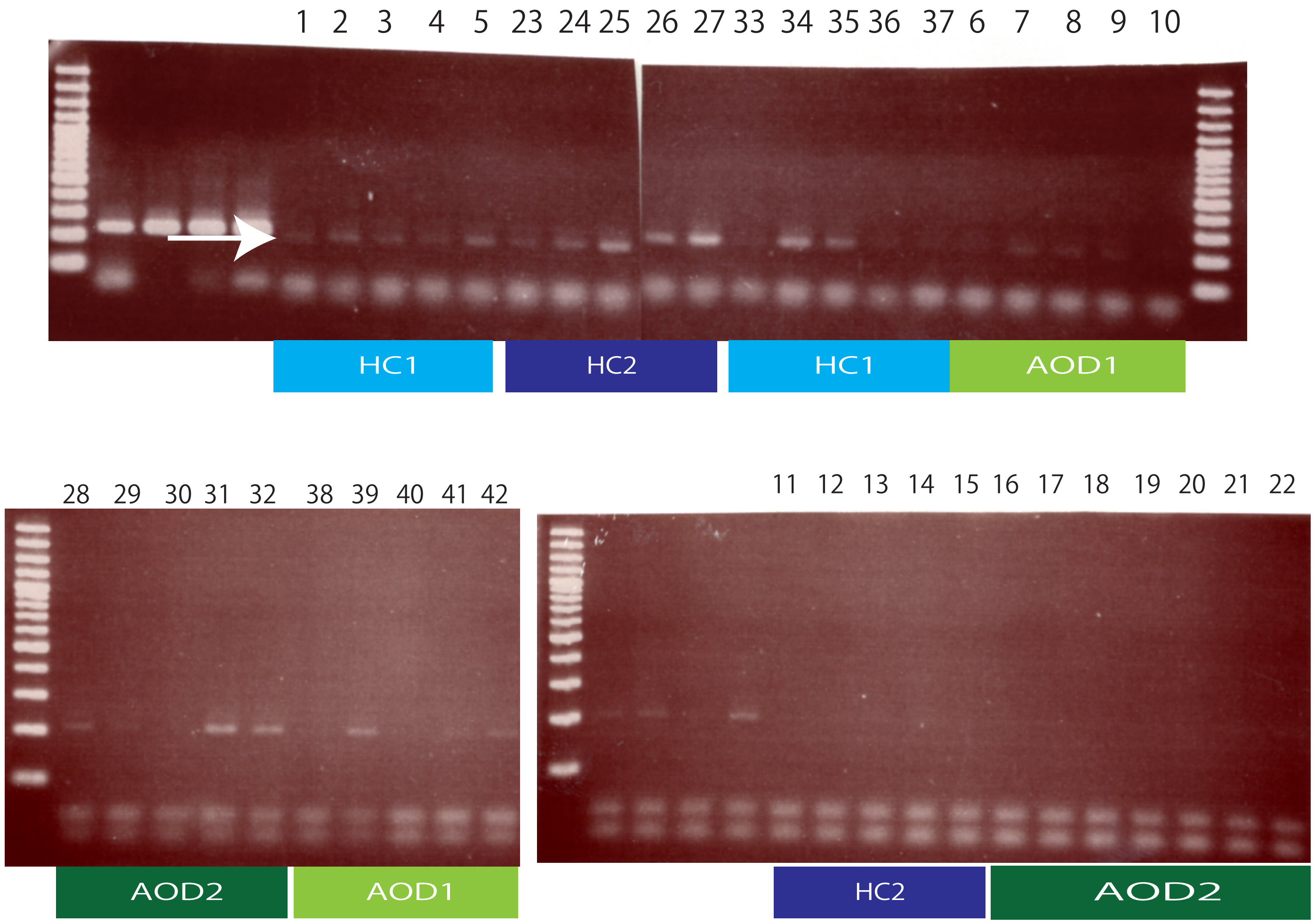

cg15871980

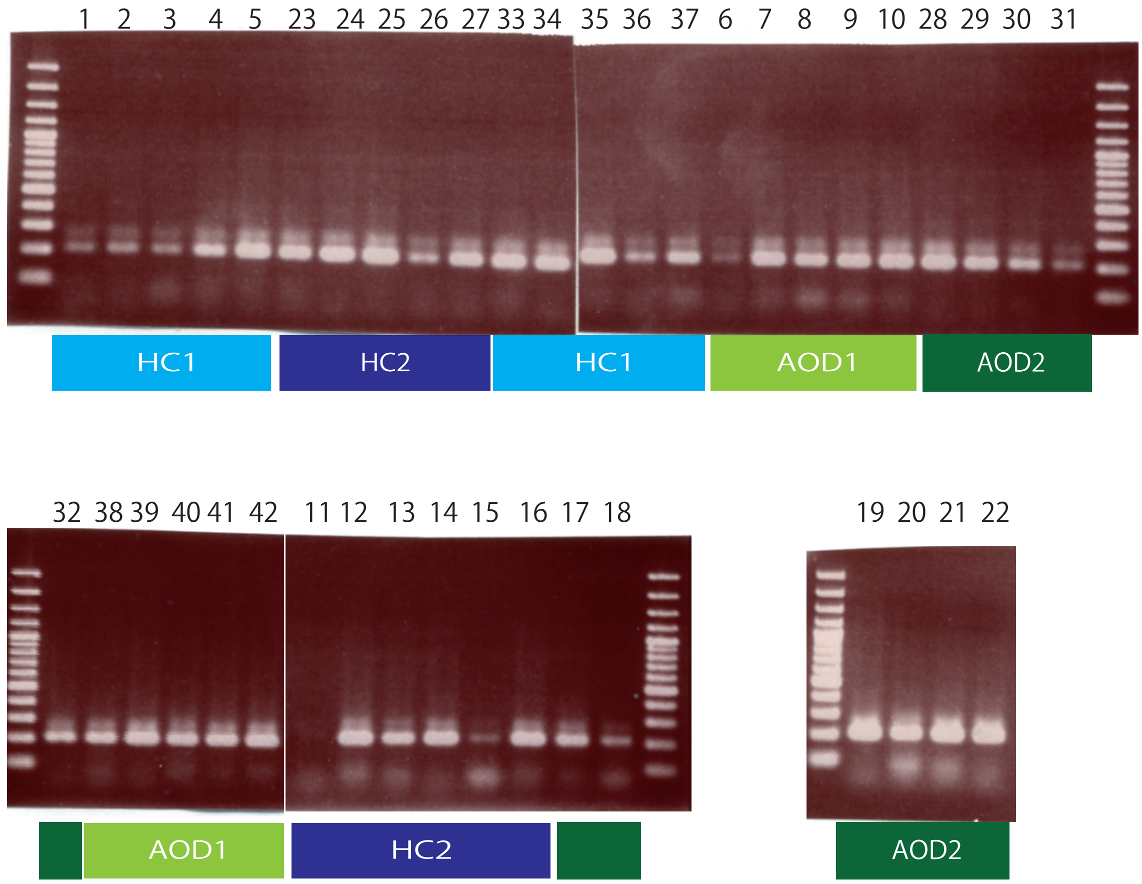

cg15794987

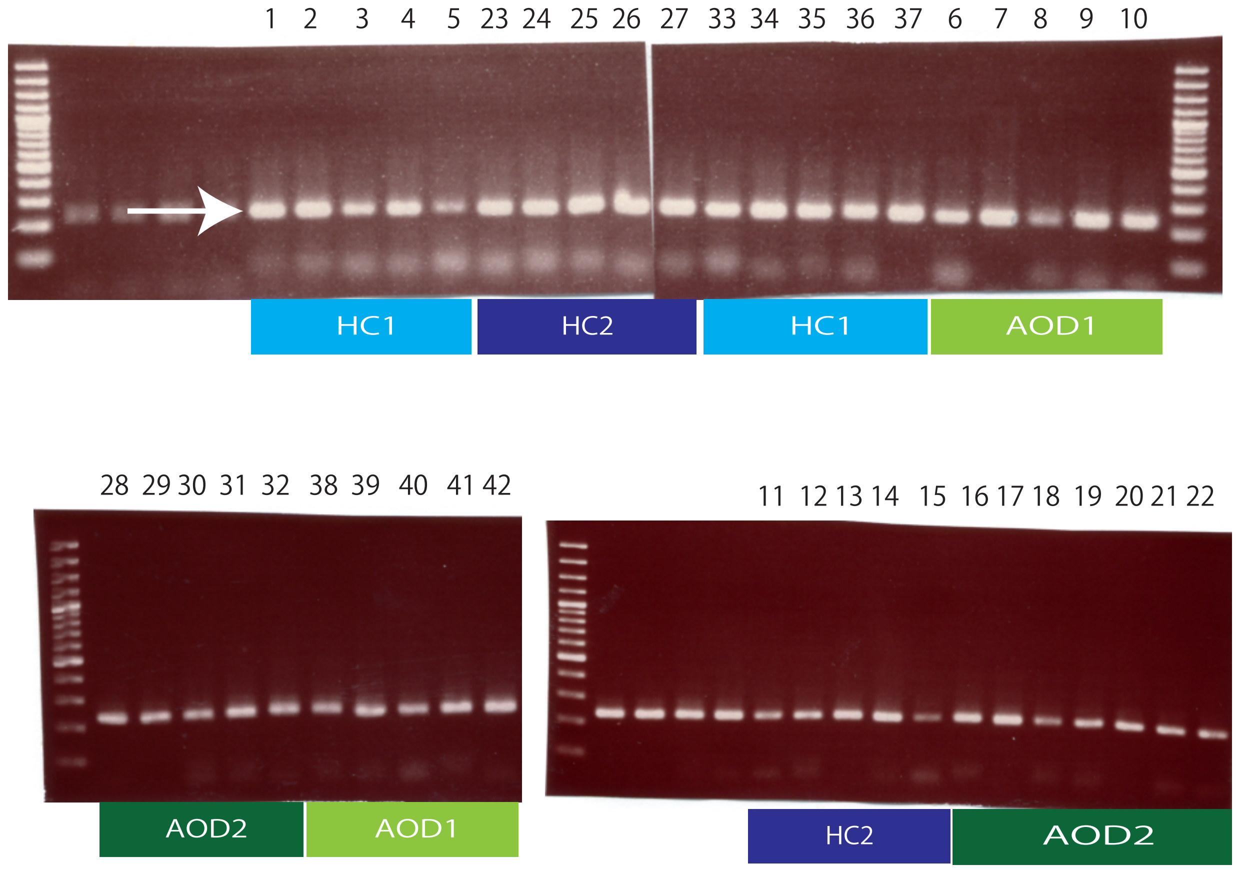

1 2 3 4 5 23 24 25 26 27 33 34 35 36 37 6 7 8 9 10
HC1
HC2
HC1
AOD1
28 29 30 31 32 38 39 40 41 42
AOD2
AOD1
11 12 13 14 15 16 17 18 19 20 21 22
HC2
AOD2

Figure S12 A summary of statistical strategy

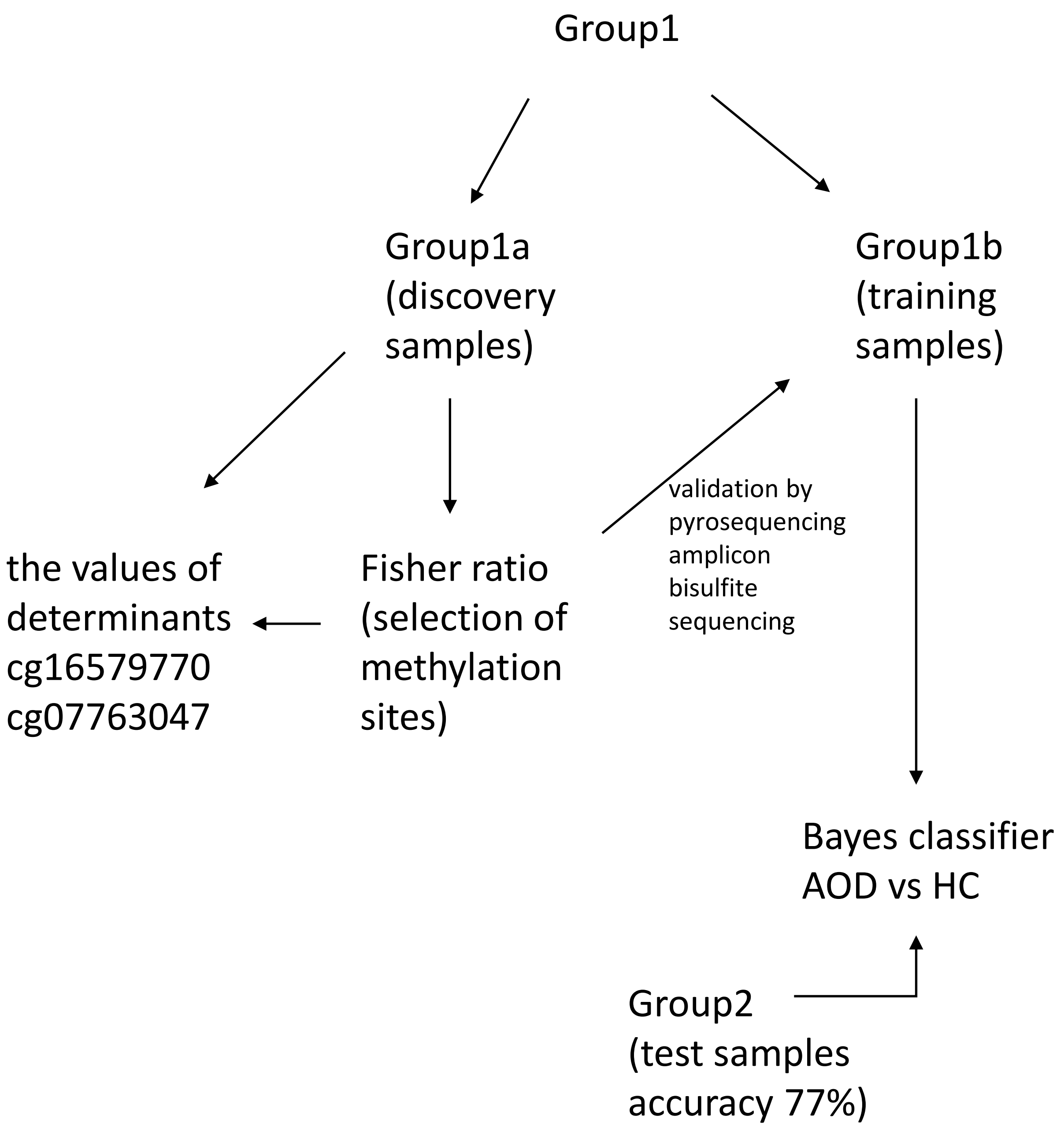

Supplement: Supplementary file 2 — Supplementary Information 2. [file 41598_2021_81758_MOESM2_ESM.pdf]
